# MaxEnt and Marxan modeling to predict the potential habitat and priority planting areas of *Coffea arabica* in Yunnan, China under climate change scenario

**DOI:** 10.3389/fpls.2024.1471653

**Published:** 2024-11-28

**Authors:** Xia Li, Zihao Wang, Shaoqiang Wang, Zhaohui Qian

**Affiliations:** ^1^ College of Environmental Science and Engineering, Tongji University, Shanghai, China; ^2^ Foreign Environmental Cooperation Center, Ministry of Ecology and Environment, Beijing, China; ^3^ Hubei Key Laboratory of Regional Ecology and Environmental Change, China University of Geosciences, Wuhan, China; ^4^ Key Laboratory of Ecosystem Network Observation and Modeling, Institute of Geographic Sciences and Natural Resources Research, CAS, Beijing, China; ^5^ Institute of Advanced Studies, China University of Geosciences, Wuhan, China

**Keywords:** Arabica coffee, MAXENT model, Marxan model, main environmental variable, potential habitat, priority planting area

## Abstract

**Introduction:**

*Coffea arabica* (Arabica coffee) is an important cash crop in Yunnan, China. Ongoing climate change has made coffee production more difficult to sustain, posing challenges for the region’s coffee industry. Predictions of the distribution of potentially suitable habitats for Arabica coffee in Yunnan could provide a theoretical basis for the cultivation and rational management of this species.

**Methods:**

In this study, the MaxEnt model was used to predict the potential distribution of suitable habitat for Arabica coffee in Yunnan under current and future (2021-2100) climate scenarios (SSP2-4.5, SSP3-7.0, and SSP5-8.5) using 56 distributional records and 17 environmental variables and to analyze the important environmental factors. Marxan model was used to plan the priority planting areas for this species at last.

**Results:**

The predicted suitable and sub-suitable areas were about 4.21×10^4^ km^2^ and 13.87×10^4^ km^2^, respectively, accounting for 47.15% of the total area of the province. The suitable areas were mainly concentrated in western and southern Yunnan. The minimum temperature of the coldest month, altitude, mean temperature of the wettest quarter, slope, and aluminum saturation were the main environmental variables affecting the distribution of Arabica coffee in Yunnan Province. Changes in habitat suitability for Arabica coffee were most significant and contracted under the SSP3-7.0 climate scenario, while expansion was highest under the SSP5-8.5 climate scenario. Priority areas for Arabica coffee cultivation in Yunnan Province under the 30% and 50% targets were Pu’er, Xishuangbanna, Honghe, Dehong, and Kunming.

**Discussion:**

Climate, soil, and topography combine to influence the potential geographic distribution of Arabica coffee. Future changes in suitable habitat areas under different climate scenarios should lead to the delineation of coffee-growing areas based on appropriate environmental conditions and active policy measures to address climate change.

## 1 Introduction

Coffee as one of the world’s three major beverage crops (Wu et al., 2016), ranks second only to petroleum as the world’s second-largest traded commodity (Eriyagama et al., 2013). Coffee plants are currently cultivated in over 80 countries globally, with the coffee industry significantly shaping the agricultural economies of major coffee-producing nations (ICO, 2022), providing livelihoods for millions of people directly or indirectly dependent on coffee. In Asia, China ranks as the fourth largest coffee exporter after Vietnam, Indonesia, and India (ICO, 2022), accompanied by an annual foreign exchange income of $532 million (Zhang et al., 2021b). Yunnan Province has a coffee cultivation history of over 100 years, with coffee planting area, production, and agricultural output value accounting for over 98% of the national total (Ma et al., 2022). In 2021, the planting area and green bean production in Yunnan Province accounted for 0.82% and 1.08% of the global total (Yunnan Provincial Department of Agriculture and Rural Affairs, 2022), making it a unique and dominant industry in China’s Yunnan Province. Coffea arabica (Arabica coffee) is the predominant variety in the region and globally (Rigal et al., 2020a), primarily used for specialty coffee, with high economic value (Fain et al., 2018), serving as the primary cash crop for local growers. In 2021, the proportion of specialty coffee in Yunnan Province was only 8%, but it increased to 22.7% by 2023, indicating a shift towards specialty coffee as the direction of development in Yunnan Province’s coffee industry (Yunnan Provincial Department of Agriculture and Rural Affairs, 2023). However, coffee cultivation will transform forests, and the future of Yunnan’s forests will likewise be highly influenced by the intensity of management (Kebebew and Ozanne, 2022). Despite commitments from the private sector, including coffee traders, aimed at eliminating deforestation from operations or supply chains, zero deforestation policies may not be sufficient to have a wider impact on their own, due to leakage, lack of transparency and traceability, selective adoption, and the marginalization of smallholder farmers (Lambin et al., 2018). Therefore, the promotion of coffee cultivation likewise needs to be integrated with public policies put in place by the government, especially spatial planning policies, to harmonize forest conservation with community interests.

Climate is a primary abiotic factor influencing species distribution (Bezeng et al., 2017). Climate change affects plant growth, geographical distribution, and population size (Alan Pounds et al., 2006), posing challenges to global agriculture and forestry, including the coffee industry. Arabica coffee and Robusta coffee (*Coffea canephora*) are the most cultivated species, with relatively narrow ranges of bioclimatic parameters (Teketay, 1999). Among them, Arabica coffee is particularly sensitive to climate (Davis et al., 2012), and coffee planted under marginal bioclimatic conditions is more susceptible to environmental stress (Fain et al., 2018). Continued climate change may exacerbate existing issues and create new challenges for the coffee industry, making production more difficult to sustain (Reyer et al., 2017). Climate change due to strong emissions will reduce the global area suitable for coffee cultivation by about half, and higher temperatures may reduce Arabica coffee yields. In Asia, some forested areas may instead become more suitable (Bunn et al., 2015). Previous studies have shown the negative impacts of climate change on coffee production in major coffee-producing countries such as Brazil, Ethiopia, and India (Koh et al., 2020; Adane and Bewket, 2021; Byrareddy et al., 2024). Understanding the effects of climate change on the biological distribution of Arabica coffee in Yunnan Province, China, and how Arabica coffee adapts to changing climatic conditions is of significant importance for the future ecological sustainability of the coffee industry in this region (Wang et al., 2019).

Species Distribution Models (SDMs) use environmental data for sites of occurrence (presence) of a species to predict a response variable to predict whether the environmental conditions are suitable for the species to persist meaning that the species is expected to occur (Araújo and Peterson, 2012). Since BioClim as the first tool to be widely used, SDMs have been widely applied to model biological responses to climate change (Booth et al., 2014). The Maximum Entropy (MaxEnt) model is a widely used SDM technique employed to estimate species distribution probabilities based on the MaxEnt principle. It relies on a statistical procedure using real observation data of species presence or abundance to infer potential suitability based on environmental characteristics, thus representing spatial suitability for species occurrence based on the variables used (Phillips et al., 2004; Phillips and Dudík, 2008). The MaxEnt model assumes that the relationship between species distribution and environmental variables maximizes entropy, and even if the information on species distribution data and the environmental variables in the distribution area is incomplete, it can still make relatively accurate predictions of the potential distribution areas of the species with good stability, and the predictions are basically in line with the actual distribution of the species, especially in the preliminary stage of the study, which provides valuable insights into the potential patterns of the species distribution (Xu et al., 2024). Compared to models such as BIOCLIM and DOMAIN, the MaxEnt model has a significant advantage of higher prediction accuracy, smaller confidence intervals, and is more stable and less affected by the random variable (Hernandez et al., 2006; Giovanelli et al., 2010; Hijmans, 2012; Duan et al., 2014). It is also capable of handling multiple types of environmental data (e.g., topography, soils, etc.) in addition to climate variables, comparing with the Climate Change Adaptation Modeler of TerrSet, to predict different climate scenarios, which provides a flexible tool to respond to diverse challenges (Ngoy and Shebitz, 2020; Lessmann et al., 2014; Han et al., 2024). Currently, the MaxEnt model has been widely used in plant protection-oriented species distribution studies (He et al., 2021). In the case of cash crops, several studies have applied the model to the suitability of corn, goji berries, citrus, coffee, etc (Davis et al., 2012; Fitzgibbon et al., 2022; Li et al., 2024; Lin et al., 2022; Zhang et al., 2021b; Cassamo et al., 2023). In China, although Zhang et al. (2021a) compared the performance of the AHP-GIS method with that of the MaxEnt model using default parameters and concluded that the latter is a more suitable tool for simulating the potential distribution of Arabica coffee in Yunnan, however, MaxEnt has been criticized as giving a biased representation of suitable climates if the parameters are not chosen carefully (Bunn et al., 2015). Therefore, the parameters need to be debugged and optimized to apply to a particular species when using the model.

Systematic conservation planning is a method frequently employed internationally for biodiversity conservation and determining priority conservation areas. It involves systematically protecting the biodiversity features of an entire area, and it is a purposeful, effective investment (Segan et al., 2011). Based on the complementarity principle, the Marxan model uses simulated annealing algorithms to identify the optimal set of planning units through iterative operations and repeated selections (Lehtomäki and Moilanen, 2013). It allows for the comparison of different potential conservation areas based on user-defined goals and costs and determines the regional set that most effectively achieves its objectives (Cudlín et al., 2020), addressing the minimum set problem in conservation planning (Smith et al., 2010). While the MaxEnt model emphasizes the suitability of species distribution based on natural environmental variables such as temperature, precipitation, and soil, the Marxan model identifies priority areas that reduce conflicts between humans and ecosystems and minimizes human impacts on species, thus considering more socio-economic factors than the MaxEnt model (Yu et al., 2023). By combining the MaxEnt and Marxan models, relevant management costs can be minimized. Simulating the spatial distribution of coffee habitat as a feature to support a systematic conservation planning model can lead to spatial planning that maximizes benefits and minimizes land costs.

This study utilized multi-source occurrence record geographic location data, current climate variables under different Shared Socioeconomic Pathways (SSPs) from CMIP6, as well as environmental data such as terrain and soil, to model the potential suitable habitat distribution of Arabica coffee in Yunnan Province, China using the MaxEnt model after parameter optimization with the Kuenm R package, including the geographical distribution characteristics under current climate conditions and the main environmental variables influencing it. Future potential geographical distribution, spatial changes, and centroid migration trajectories under different climate scenarios were also predicted. Finally, using the distribution probability values of Arabica coffee as the conservation feature and land use/land cover and natural protected areas data as costs, the Marxan model was employed to identify priority planting areas for Arabica coffee in Yunnan Province. The results of the study provide a sustainable and implementable reference for spatial planning and agricultural policies for local government decision-makers and smallholder farmers to promote the cultivation of Arabica coffee.

## 2 Materials and methods

### 2.1 Collection and processing of sample distribution points

**Figure 1:**
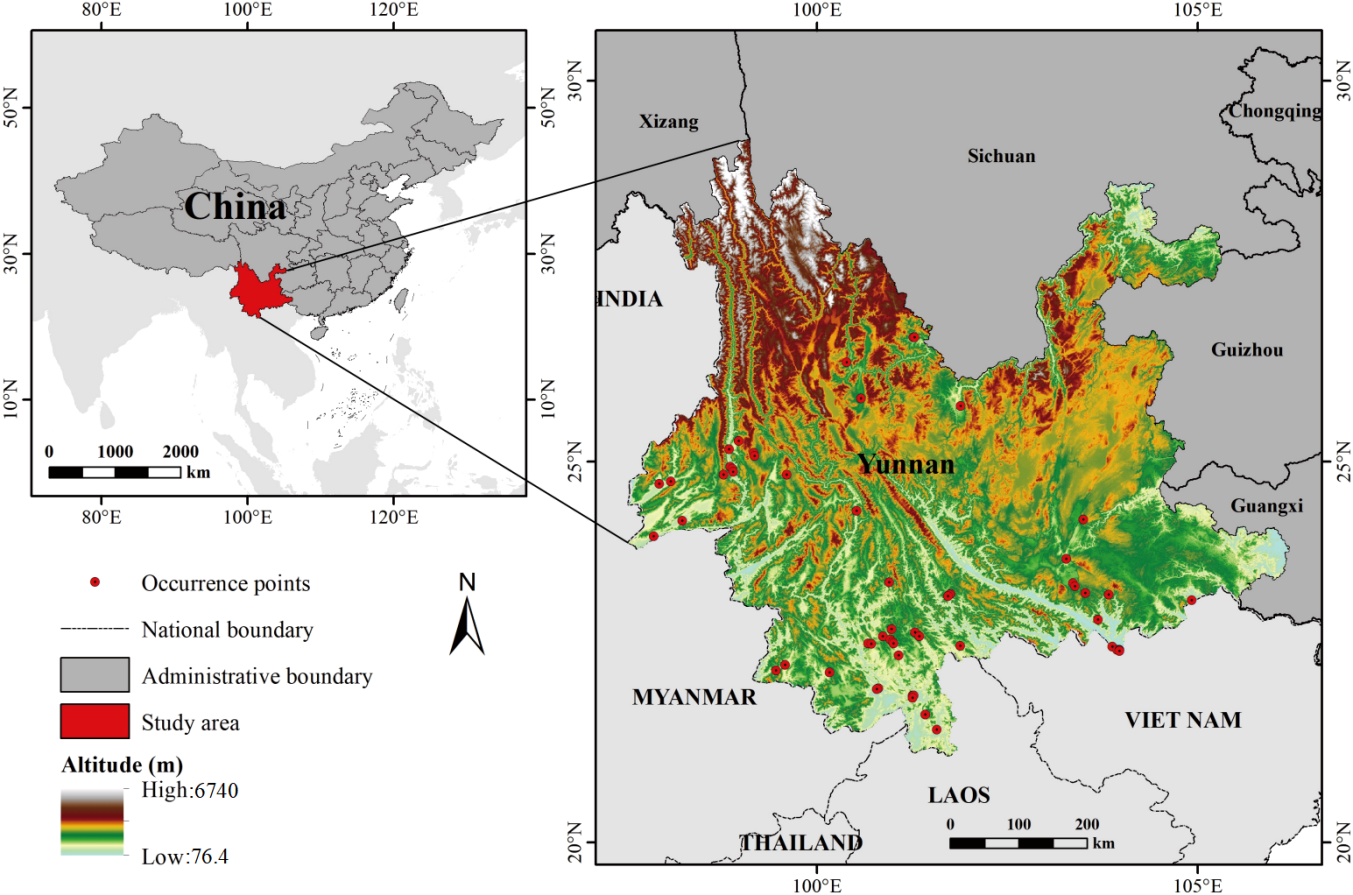
Sample distribution of *Coffea arabica* in the study area.

The distribution location data of *Coffea arabica* in Yunnan Province used in this study were obtained from the Global Biodiversity Information Facility (GBIF, https://www.gbif.org), Naturalist (https://www.inaturalist.org), Biological Plant Specimen Museum of the Chinese Academy of Sciences (https://pe.ibcas.ac.cn), Chinese Virtual Herbarium (CVH, https://www.cvh.ac.cn) and relevant papers on Yunnan Province Coffee (Ge et al., 2023; Hao et al., 2022; Li et al., 2021, 2023; Lu et al., 2022; Rigal et al., 2020a, 2020b; Wan et al., 2024; Yang et al., 2022) collected from the Web of Science (WOS, https://www.webofscience.com), totaling 331 sample points. All occurrence records especially those lacking precise geographic coordinates were revised with specific latitude and longitude information using Google Earth. To minimize spatial autocorrelation between sample points and reduce errors caused by model overfitting due to sampling bias, duplicate records were removed using ENMTools 1.4.4, ensuring that only one point was retained per grid cell, and each remaining point was moved to the center of its grid cell (Ma et al., 2024; Warren et al., 2021). After the aforementioned processing, a total of 56 valid species sample distribution records were adopted (**Figure 1**).

### 2.2 Selection of environmental variables

To simulate the current and future suitable habitat for coffee cultivation in Yunnan Province, a total of 32 environmental variables potentially influencing the distribution of Arabica coffee was selected based on the previous study (Zhang et al., 2021b). These factors include climate, terrain, and soil variables (**Table 1**). For the current and future (from 2021 to 2100) climate data, we retrieved raster layers for future climate projections under three Shared Socio-economic Pathways (SSPs), namely SSP2–4.5(Upgrade of scenario RCP4.5 from SSP2 for the medium forcing scenario), SSP3-7.0 (Additional scenario RCP7.0 emission pathways based on SSP3 for a rocky road) and SSP5–8.5 (Upgrade of scenario RCP8.5 from SSP5 for high forcing scenario) for the bioclimatic variables (Lenoir et al., 2008; Jia et al., 2024). The layers were extracted from the sixth version of Model for Interdisciplinary Research on Climate (MIROC6) which was retrieved from the sixth phase of the Coupled Model Intercomparison Project (CMIP6) in WorldClim (https://www.worldclim.org/data/cmip6/cmip6_clim30s.html). The model has shown good performance in climate characterization (Tian et al., 2021), particularly in southern China, and has been widely used in the simulation of species distribution models in this region as well as in other similar climatic zones (Li et al., 2022; Coulibaly et al., 2023; Oduor et al., 2023). Terrain data were obtained from the NASA Digital Elevation Model (NASA EARTHDATA, https://search.earthdata.nasa.gov) with a spatial resolution of 30 meters. Elevation, slope, and aspect were extracted for the study area using ArcGIS 10.8. Soil data were sourced from the Harmonized World Soils Database version 2.0 (HWSD 2.0, https://gaez.fao.org/pages/hwsd), providing information on the morphological, chemical, and physical properties of soils at approximately 1 km resolution. The soil characteristics of the topsoil layer (D1) of the soil mapping units with the highest share proportion were utilized. All environmental variables were resampled to a spatial resolution of 30 arc-seconds (ca. 1 km^2^ at the equator) and converted into the appropriate format using ArcGIS 10.8.

**Table 1:** Environmental variables used to model the potential distribution of Arabica coffee.

Category	Variable	Description	Unit
Bioclimatic variables	bio1	Annual mean temperature	°C
	bio2	Mean diurnal range	°C
	bio3	Isothermality (bio2/bio7) (×100)	–
	bio4	Temperature seasonality (standard deviation ×100)	–
	bio5	Max temperature of the warmest month	°C
	bio6	Min temperature of the coldest month	°C
	bio7	Temperature annual range (Bio5-Bio6)	°C
	bio8	The mean temperature of the wettest quarter	°C
	bio9	The mean temperature of the driest quarter	°C
	bio10	The mean temperature of the warmest quarter	°C
	bio11	The mean temperature of the coldest quarter	°C
	bio12	Annual precipitation	mm
	bio13	Precipitation of the wettest month	mm
	bio14	Precipitation of the driest month	mm
	bio15	Precipitation seasonality (coefficient of variation)	mm
	bio16	Precipitation of the wettest quarter	mm
	bio17	Precipitation of the driest quarter	mm
	bio18	Precipitation of the warmest quarter	mm
	bio19	Precipitation of the coldest quarter	mm
Terrain variables	altitude	Altitude	m
	slope	Slope	°
	aspect	Aspect	°
Soil variables	sand	Topsoil sand fraction	% wt
	silt	Topsoil silt fraction	% wt
	clay	Topsoil clay fraction	% wt
	org_carbon	Organic carbon content	g/kg
	ph_water	pH Value (H_2_O)	-log(H^+^)
	total_n	Total nitrogen content	g/kg
	cec_soil	Cation exchange capacity soil	cmol/kg
	alum_sat	Aluminum saturation	% ECEC
	tcarbon_eq	Calcium carbonate	% weight
	gypsum	Gypsum content	% weight

**Figure 2:**
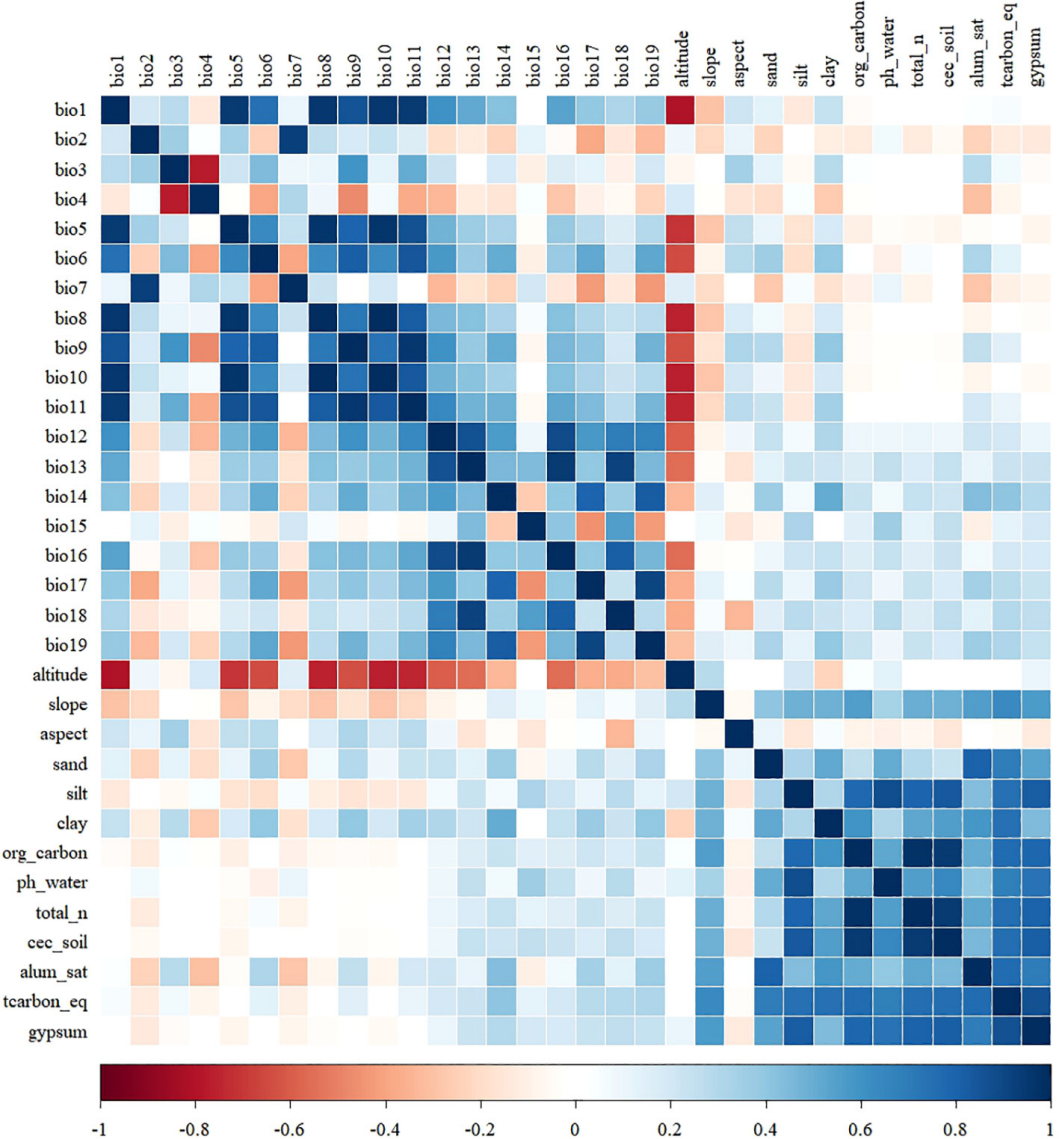
Result of Pearson’s correlation coefficient.

Species distribution models (SDMs) may be influenced directly or indirectly by the predictive variables used, due to factors such as approximation, causality, or correlation among them (Zhang et al., 2020). To mitigate issues such as multicollinearity, autocorrelation, and redundancy of environmental variables that may lead to model overfitting (Burgos et al., 2020), we first conducted a Pearson correlation coefficient analysis to retain factors within each environmental variable type with correlation coefficients |r|< 0.85 as illustrated in **Figure 2**. Result of Pearson’s correlation coefficient. Subsequently, all environmental factors were inputted into a preliminary MaxEnt model with default parameters, and factors with low or zero contribution rates in the results were removed. Finally, 17 key factors (bio3, bio4, bio6, bio7, bio8, bio12, bio14, bio15, bio17, bio18, altitude, slope, aspect, alum_sat, clay, pH_water, tcarbon_eq) were identified as the environmental variables for predicting the distribution of Coffea Arabica in Yunnan Province. The results of the preliminary experiment are presented in **Supplementary Table S1**.

### 2.3 Establishment, optimization, and evaluation of MaxEnt model

The principle of maximum entropy refers to the full consideration of known information when inferring the unknown probability distribution, mainly based on Shannon’s information entropy theory, which decreases when the total amount of information increases (Allahverdyan et al., 2021). Under the premise of ensuring the inclusion of existing information, when the entropy is maximized, it can be determined that the least amount of unknown information is included, thus reducing the uncertainty brought by unknown information. Assuming a point exists in a multidimensional natural environmental space, associated with multiple environmental factor parameters, it becomes possible to extract the environmental factor parameters linked to all distribution points of a species within this space. By applying specific algorithms, the ecological requirements of the species can be inferred, and the results can then be projected onto different temporal and spatial geographic regions to predict the potential distribution range of the species in a particular area (Elith et al., 2011). A formal explanation of the MaxEnt model based on this principle can be found in **Supplementary Material**.

Phillips et al. (2004) developed the MaxEnt software for predicting species’ potential distributions on the Java platform. This software establishes constraints based on the environmental variable characteristics of actual geographical distribution points of the species and subsequently explores possible distributions under the principle of maximum entropy. The probability distribution of species presence at maximum entropy is anticipated to closely align with the species’ actual distribution. The process for utilizing the MaxEnt model involves several key steps: first, the collected occurrence data is inputted in CSV format into the “Samples” module, organized by species name, longitude, and latitude. Next, relevant environmental factor variables are standardized in terms of boundaries, coordinate systems, and raster sizes using GIS, and then converted into ASC layer format for input into the “Environmental layers” module, where environmental variables can be designated as either discrete or continuous. Following this, the user sets the number of iterations (typically 10) or the proportion of validation data (20%-30%) and determines whether to generate response curves for evaluating model accuracy, as well as whether to employ the jackknife method for selecting dominant environmental factors (Phillips, 2017). Finally, the output results are interpreted to derive insights from the model’s predictions.

Species distribution models (SDMs) utilize options and algorithm settings that influence model complexity to describe the relationship between occurrence data and environmental variables. However, default settings often lead to overly complex models and overfitting of training data, resulting in distorted estimates of suitability and poor transferability to other locations or times (Soley-Guardia et al., 2024). Therefore, before using the MaxEnt model to predict the suitable habitat for Arabica coffee in Yunnan Province, we optimized it using the R package kuenm. The analysis began with input data, including thoroughly filtered and sparse species occurrence records to calibrate a complete event set, randomly partitioned into training event subsets and a 30% subset of events for testing candidate models (Cobos et al., 2019), along with environmental variables for creating candidate models. Additionally, a set of Arabica coffee occurrence records from Yunnan Province, collected from papers in WOS that were not used in the calibration process, was used as fully independent occurrence data to test the final model. This dataset had a different source from the other datasets and had no spatial autocorrelation with the calibration data. A range of 0.1 to 4, with an interval of 0.1, for the regularization multiplier (RM), and feature combinations (FC) including linear (L), quadratic (Q), product (P), threshold (T), and hinge (H), were cross-combined to explore parameter interactions. A total of 1240 candidate models were evaluated, reflecting all combinations of 40 regularization multiplier settings, 31 feature class combinations, and 1 distinct set of environmental variables. Model performance was assessed based on statistical significance (Partial ROC), omission rates (OR), and the Akaike information criterion corrected for small sample sizes (AICc). Finally, significant models with omission rates ≤5% and Delta_AICc=0 were selected, indicating the best model mobility from known distribution areas to predicted areas and effectively avoiding overfitting (Warren et al., 2014; Warren and Seifert, 2011).

### 2.4 Species habitat suitability prediction

The optimal parameter combination was inputted into MaxEnt for modeling and the average value was chosen as the model output result after 10 repetitions. The impact of environmental factors on the model was determined using the jackknife test method, and predictive charts were plotted to evaluate the importance of environmental variables. During the model-building process, the accuracy of the model was verified using the Area Under the Curve (AUC) value in the jackknife test. AUC represents the area under the Receiver Operating Characteristic (ROC) curve and theoretically ranges from 0.5 to 1. Higher values indicate higher predictive accuracy, with values between 0.9 and 1 indicating excellent predictive performance. The model generated logical output (LO) values ranging from 0 to 1, representing the suitable index for different regions. The selection of thresholds is crucial for predicting different levels of suitability areas, thus affecting the calculation of different suitable regions. We selected the Maximum Training Sensitivity plus Specificity (MTSS) and the balance of training omission, predicted area, and threshold value (TPT) as classification thresholds for suitable and less suitable areas, respectively, to reclassify LO. As a result, the potentially suitable habitat for Arabica coffee in Yunnan Province was classified into three levels: suitable, less suitable, and unsuitable habitats (Clark et al., 2014; Liu et al., 2013).

### 2.5 Future potential habitat prediction under different climate scenarios

By altering future bioclimatic variables, this study created potential habitats for Arabica coffee in Yunnan Province under three different Shared Socioeconomic Pathways (SSPs): SSP2-4.5, SSP3-7.0 and SSP5-8.5, for four future periods (2021-2040, 2040-2060, 2060-2080 and 2080-2100, i.e., 2030s, 2050s, 2070s and 2090s). Using the Distribution Changes Between Binary SDMs tool in SDM Toolbox v2.6 within ArcGIS 10.8, this study further simulated the spatial pattern changes including range expansion, unoccupied areas, no change, and range contraction to analyze the impact of climate change on the size of potential habitats for the species (Brown, 2014). Simultaneously, considering the suitable areas for specific scenarios and periods as a whole, they were simplified into vector particles, with changes in the central point position reflecting the trend of habitat shifts due to climate change. The Centroid Changes tool was used to calculate the coordinate changes and migration distances of current and future suitable habitats (Liang et al., 2021).

### 2.6 Construction of the Marxan model

The Marxan model is primarily designed to address the minimum set cover problem which aims to protect the minimum number of conservation targets that must be protected at the lowest cost and make it easier to establish protected areas without disrupting existing community continuity (Klein et al., 2009). The Marxan software uses a minimum coverage set model and incorporates a simulated annealing algorithm to complete the protected area siting analysis with the following operational objective function (Watts et al., 2009). Considering that the government controls land use through spatial planning, this study defines the township administrative area as the smallest planning unit which reflects the human-land relationship in coffee cultivation for convenient management. After removing administrative regions with excessively small areas, the study area is divided into a total of 1392 planning units. Using the zoning statistics tool in ArcGIS 10.8, the existence probability of the target species in each planning unit under current climatic conditions is calculated to construct a species distribution feature matrix.


$$\begin{array}{c} \sum_{PU_{s}}Cost+BLM\sum_{PU_{s}}Boundary+\sum_{Convalue}SPF\times Penalty\\ +CostThresholdPenalty\left(t\right) \end{array}$$

$\sum_{PU_{s}}Cost$ is a cost factor that represents the total cost of the selected planning units in the program. We established its values for the ratio of the area of land use/land cover (LULC) types where coffee cultivation is prohibited and the area of nature reserves to the total area of the planning unit. The LULC data is derived from the 30-meter annual China Land Cover Dataset (CLCD) (Yang and Huang, 2021), selecting LULC types such as cropland, water, snow/ice, barren, impervious, and wetland for the year 2019. Natural protected area data are obtained from Key Biodiversity Areas (KBA, https://www.keybiodiversityareas.org), The World Database on Protected Areas (WDPA, https://www.protectedplanet.net/), and the China Natural Protected Area Specimen Resource Sharing Platform (PAPC, https://www.papc.cn). BLM is the boundary length modifier, $\sum_{PU_{s}}Boundary$ is the boundary factor, characterizes the total boundary length of the selected planning units in the scheme, and together they can regulate the compactness of the selected planning units; $\sum_{Convalue}SPF\times Penalty$ is a penalty factor for species conservation, SPF is the penalty for a single planning unit that does not accomplish the protection goal, and Penalty is the penalty coefficient. By adjusting the size of the SPF value, the size of the objective function can be controlled when the protection goal is not achieved, so that the selected planning units can accomplish the protection goal as much as possible. Based on the requirements of the Yunnan Province Provincial Government’s Action Plan for Agricultural Modernization, the conservation target is set at 30% and the forward target is set at 50%. CostThresholdPenalty(t) is a penalty factor when the upper limit of the cost threshold is exceeded.

**Figure 3:**
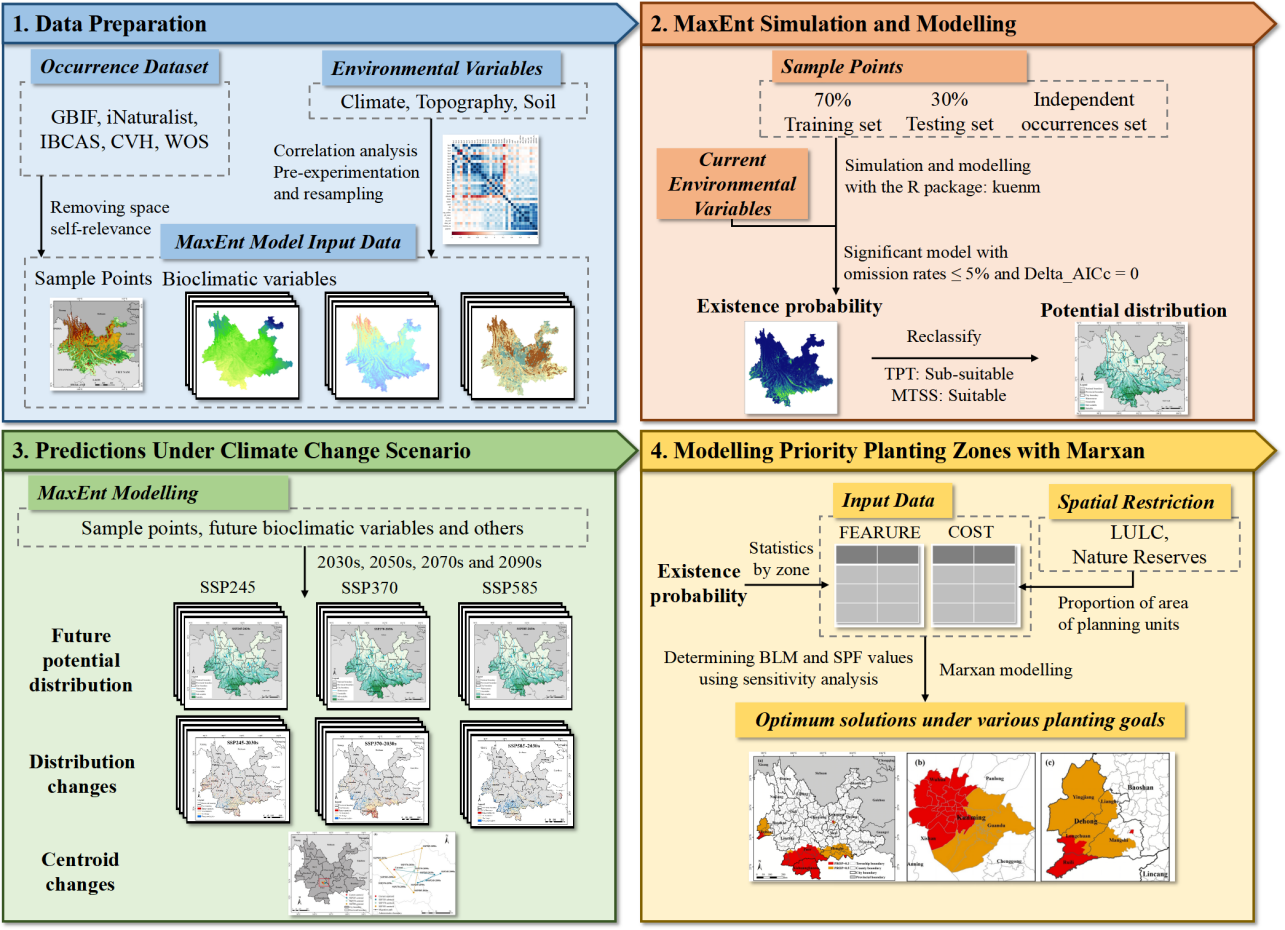
Conceptual and methodological flowchart.

The Species Penalty Factor (SPF) and Boundary Length Modifier (BLM) are used in the model to balance the conservation of species in planning and the length of protected area boundaries. Sensitivity analysis is conducted by plotting sensitivity curves to find the most suitable values for BLM and SPF. Finally, BLM = 1.6 and SPF = 1.6 are determined, and the model is iterated 100 times to obtain the optimal solution for planning units. The specific roles of the parameters and the sensitivity analysis process for determining them are detailed in the **Supplementary Material**. The conceptual and methodological flowchart of this study is shown in **Figure 3**.

## 3 Results

### 3.1 Model optimization and accuracy assessment

Based on 56 species distribution records and 17 environmental variables, the potential suitable habitat distribution of Arabica coffee in Yunnan Province was predicted. Among the 1240 model results, combinations that were statistically significant and met the criteria for model omission rates and AICc were selected: RM set to 1.5 and FC combination set to QTH. The model performance is shown in **Supplementary Figure S4**. The model was run 10 times using this combination setting, resulting in an average training AUC for the replicate runs of 0.935 ± 0.013 (**Supplementary Figure S5**). Among these runs, the model with the highest test gain value both exceeding 0.9 had an AUC of 0.938 for the training data and 0.968 for the test data (**Supplementary Figure S6**). This indicates excellent predictive accuracy of the model, with high confidence in the predicted results.

### 3.2 Main environmental variables

The importance of environmental variables is mainly assessed by the percent of contribution (PC) and permutation importance (PI) indices where higher values indicate greater importance (Zhang et al., 2021a). Based on the MaxEnt simulation results for Arabica coffee, 7 environmental variables had very small contributions or permutation importance values (<1%), hence only 10 dominant environmental variables are provided (**Table 2**. Dominant environmental variables for the potential distribution of Arabica coffeeDominant environmental variables for the potential distribution of Arabica coffee). The top 5 factors by percent of contribution are the minimum temperature of the coldest month (bio6, 20.8%), altitude (16.8%), mean temperature of the wettest quarter (bio8, 14.7%), slope (14.6%), and alum_sat (12.2%), accounting for a cumulative contribution of 79.1%. The top 5 factors by permutation importance are altitude (32.2%), mean temperature of the wettest quarter (bio8, 19%), pH water (11.5%), temperature seasonality (bio4, 7.9%) and precipitation of driest quarter (bio17, 5.5%), accounting for a cumulative permutation importance of 76.1%. In the Jackknife plot (**Supplementary Figure S7**), when analyzing with only one variable, the altitude had the best-regularized training gain value, followed by the mean temperature of the wettest quarter (bio8), precipitation of the warmest quarter (bio18) and minimum temperature of the coldest month (bio6), all with values exceeding 0.9 which significantly higher than other factors, indicating their substantial influence on the distribution of Arabica coffee in Yunnan Province. Therefore, the minimum temperature of the coldest month (bio6), mean temperature of the wettest quarter (bio8), and altitude are the dominant factors influencing the potential distribution of Arabica coffee in Yunnan Province, while precipitation of the warmest quarter (bio18), slope and aluminum saturation (alum_sat) also play important roles.

**Table 2:** Dominant environmental variables for the potential distribution of Arabica coffee.

Variable	Percent of contribution(%)	Permutation importance (%)
bio6	20.8	0.7
altitude	16.8	32.2
bio8	14.7	19
slope	14.6	4.3
alum_sat	12.2	0.7
bio12	5	1.9
bio4	3.5	7.9
bio17	3.2	5.5
bio18	2.7	3.8
ph_water	2.2	11.5

The response curves of environmental factors reflect the dependency of suitability on variables (Zhang et al., 2021a). Based on the above results, single-factor modeling was conducted and single-factor response curves were plotted (**Supplementary Figure S8**) to study the relationship between distribution probability and major environmental variables. It is generally believed that when the distribution probability is greater than 0.5, the corresponding environmental factor is suitable for plant growth (Wang et al., 2023). The range which is suitable for the survival of Arabica coffee of the lowest temperature of the coldest month (bio6) and the mean temperature of the wettest quarter (bio8) is approximately between 5 to 10°C and 23 to 25°C. Additionally, the temperature seasonality (bio4, standard deviation ×100) should be less than 450. For precipitation, annual precipitation (bio12) above approximately 1200mm is suitable for the growth. Arabica coffee in Yunnan Province is most suitable at altitudes around 1200m with the range approximately between 680m to 1750m while the slope should be less than 9°. The distribution probability of Arabica coffee decreases with increasing aluminum saturation (alum_sat) among the soil variables, indicating that soil with high aluminum content may adversely affect coffee growth.

### 3.3 Current and future potential suitable habitat distribution

**Figure 4:**
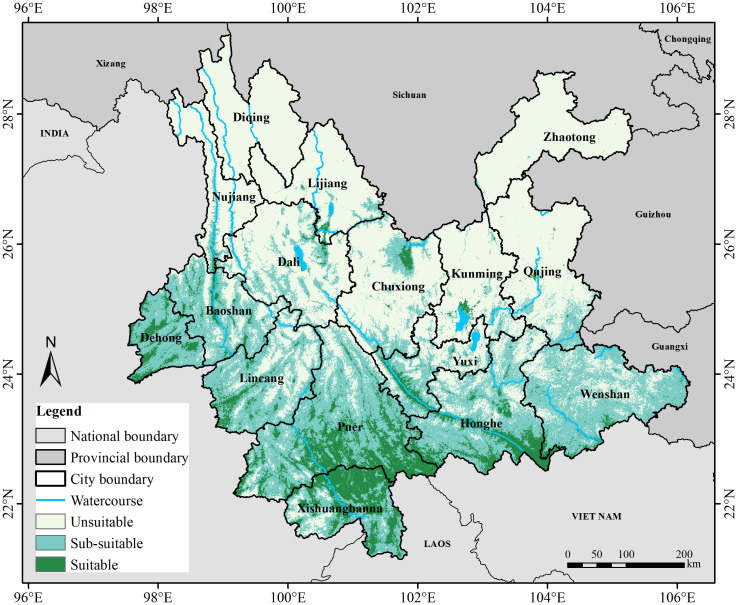
Predicted distribution of Arabica coffee in Yunnan Province under current climate condition.

Currently, the potential suitable habitat distribution area for Arabica coffee in Yunnan Province is illustrated in **Figure 4**. Both suitable and sub-suitable areas are primarily concentrated in the southern and western regions of Yunnan Province, particularly in the Hengduan Mountains region where the elevation is below 1500 meters. The predominant climatic types in these areas are the South Asian tropical and North tropical regions. The distribution areas of suitable habitats and their proportion to the total study area are presented in **Table 3**. Potential distribution areas under current climate conditions. The suitable and sub-suitable areas cover approximately 42,124.44 km^2^ and 138,732.46 km^2^, accounting for 47.15% of the total provincial area. Among them, the suitable habitat area accounts for approximately 11% of the total area of Yunnan Province. The suitable habitat areas are mainly distributed in cities (or prefectures) such as Baoshan, Dehong, Lincang, Pu’er, Xishuangbanna, Honghe, and Yuxi with their combined area accounting for about 10% of the total area of Yunnan Province. A smaller portion is distributed at the border between Dali and Lijiang, the northern part of Chuxiong, as well as Kunming and Qujing. In Pu’er and Xishuangbanna, the suitable habitat areas account for over 30% of the total city area while in Honghe and Yuxi the suitable habitat areas are mainly distributed along the Ailao Mountain range. The entire area of Wenshan has a suitable/sub-suitable habitat coverage of 75.56%, but the majority is a sub-suitable habitat with suitable habitat occupying only 4.08% of the total area.

**Table 3:** Potential distribution areas under current climate conditions.

City	Unsuitable habitat(km^2^)	Sub-suitable habitat(km^2^)		Suitable habitat(km^2^)	Percentage of suitable areas in the city(%)	Percentage of suitable areas in Yunnan Province(%)
	<TPT	TPT-MTSS		≥MTSS		
Baoshan	5225.1	12218.8		1750.5	9.12	0.46
Chuxiong	21580.1	6085.2		827.2	2.9	0.22
Dali	20562.4	7422.9		458.7	1.61	0.12
Dehong	251.1	8043.3		2952.7	26.25	0.77
Diqing	23152.4	123.1		0	0	0
Honghe	7240. 7	18165.3		6687.6	20.84	1.74
Kunming	18320.4	2278.2		403.8	1.92	0.11
Lijiang	18446. 0	1752.1		416.9	2.02	0.11
Lincang	6939.5	14713.6		2099.2	8.84	0.55
Nujiang	12369.8	1899.0		370.1	2.53	0.1
Puer	5133.2	25539.7		13715.7	30.90	3.58
Qujing	24781.4	3917.8		143.6	0.5	0.4
Wenshan	7646.1	22363.0		1276.9	4.08	0.33
Xishuangbanna	1944.1	7751.1		9331.7	49.04	2.43
Yuxi	7007.6	6272.3		1675.8	11.21	0.44
Zhaotong	22110.2	187.1		14.0	0.06	0.004

**Figure 5:**
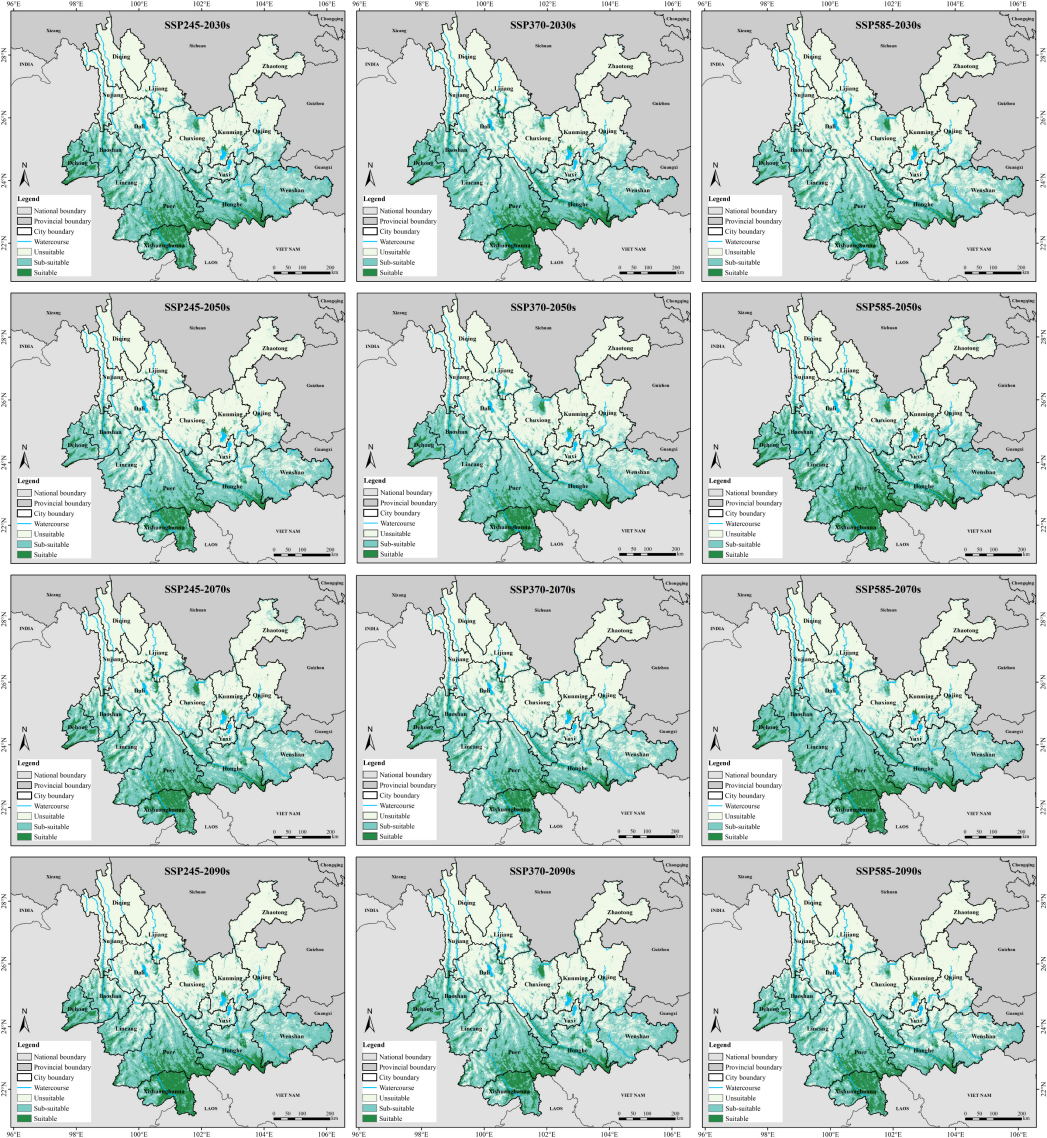
Predicted distribution of Arabica coffee in Yunnan Province under future climate scenarios (2021-2100).

Changes in bioclimatic variables were manipulated to explore the future potential suitable habitats for Arabica coffee in Yunnan Province as depicted in **Figure 5**. Predicted distribution of Arabica coffee in Yunnan Province under future climate scenarios (2021-2100). Despite the predominance of potential suitable/sub-suitable habitats in the western and southern regions of Yunnan Province during the four future periods (2021-2040, 2040-2060, 2060-2080, and 2080-2100), the characteristics of their area changes vary. The results indicate that the magnitude of changes in suitable habitat areas is generally greater than that of sub-suitable habitat areas in most cases under the same climate scenario. Except from 2060 to 2080 under the SSP2-4.5 scenario, the trends in area changes of potential suitable/sub-suitable habitats for Arabica coffee in Yunnan Province over the four future periods are consistent across different climate scenarios. Specifically, under the SSP3-7.0 scenario, the suitable habitat area decreases annually while the sub-suitable habitat area increases annually. Overall, except for a slight increase in the total area of potential suitable/sub-suitable habitats between 2021-2040, the total area shows a decreasing trend in other periods, indicating a contraction trend in potential habitats for Arabica coffee in Yunnan Province over the next 80 years under this climate scenario with suitable habitats gradually deteriorating into sub-suitable habitats. In contrast to the SSP2-4.5 and SSP3-7.0 scenarios, where the suitable/sub-suitable area shows a trend of first increasing and then decreasing during the periods of 2021-2040, 2040-2060 and 2060-2080, the suitable/sub-suitable habitat area under the SSP5-8.5 scenario significantly decreases by 32.88% between 2021-2040, then undergoes a substantial increase of 48.73% between 2040-2060 and 2060-2080, followed by a further decrease of 22.04% between 2080-2100. The area and change rates of potential suitable/sub-suitable habitats under different decade scenarios are summarized in **Table 4**. Predicted suitable areas under current and future climate conditions.

**Table 4:** Predicted suitable areas under current and future climate conditions.

Decades Scenarios		Predicted area/10^4^km^2^		Increase/decrease rate (%)	
		Suitable habitat	Sub-suitable habitat	Suitable habitat	Sub-suitable habitat
Current	–	4.39	14.52	–	–
2021-2040	SSP2-4.5	4.41	15.67	0.58	7.97
	SSP3-7.0	4.33	15.22	-1.40	4.84
	SSP5-8.5	3.14	13.88	-28.48	-4.40
2040-2060	SSP2-4.5	2.96	14.09	-32.55	-2.95
	SSP3-7.0	3.60	15.27	-17.89	5.17
	SSP5-8.5	5.31	16.17	20.93	11.35
2060-2080	SSP2-4.5	4.14	15.04	-5.72	3.62
	SSP3-7.0	3.62	14.81	-17.52	2.03
	SSP5-8.5	4.68	15.95	6.59	9.87
2080-2100	SSP2-4.5	4.84	14.57	10.29	0.37
	SSP3-7.0	3.96	15.24	-9.70	4.97
	SSP5-8.5	3.55	14.10	-19.19	-2.85

### 3.4 Potential habitat distribution changes in future climatic scenarios

**Figure 6:**
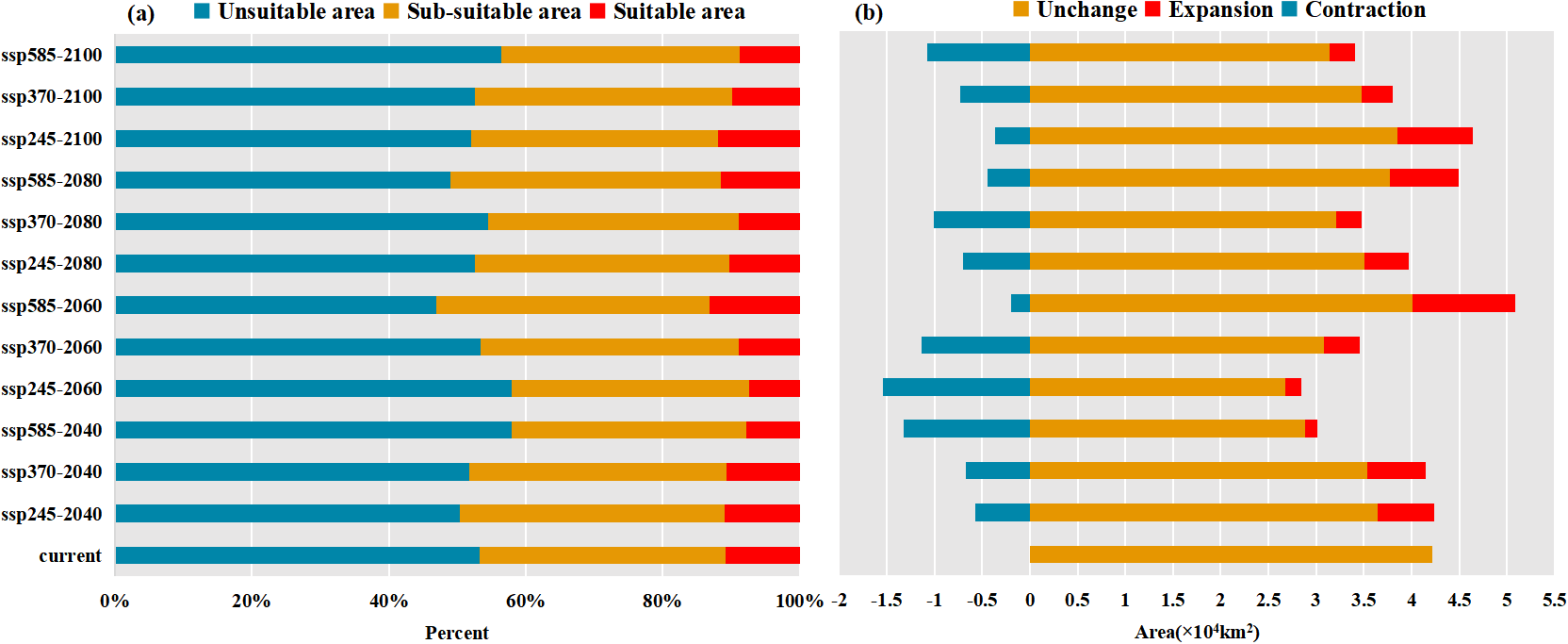
Area changes of suitable/sub-suitable habitats for Arabica coffee in Yunnan Province under future climate scenarios/periods. **(A)** Percentage of area in each suitability class; **(B)** Area change in suitable habitat.

The comparison analysis of the distribution patterns of potential suitable/sub-suitable habitats for Arabica coffee in Yunnan Province under different climate scenarios and periods compared to the current period (**Figure 6**. Area changes of suitable/sub-suitable habitats for Arabica coffee in Yunnan Province under future climate scenarios/periods. (a) Percentage of area in each suitability class; (b) Area change in suitable habitat) reveals a simultaneous expansion and contraction of suitable/sub-suitable habitats in the future. Under the SSP3-7.0 climate scenario, the suitability changes for Arabica coffee habitats are most significant, with the highest degree of contraction observed under this scenario. Conversely, the changes are least pronounced under the SSP5-8.5 climate scenario, but the expansion is highest under this scenario. Specifically, the expansion is most pronounced during the 2040-2060 period under the SSP5-8.5 scenario, with an area increase from unsuitable to suitable/sub-suitable habitats of 10,742.25 km^2^, representing a 20.31% expansion rate. Additionally, its stability is also the highest, with a stable area of 40,142.67 km^2^, accounting for 75.91% stability, indicating the most suitable growth conditions for Arabica coffee in Yunnan Province under this scenario. Conversely, the degree of retreat is highest during the 2040-2060 period under the SSP2-4.5 scenario, with an area decrease from suitable/sub-suitable to unsuitable habitats of 15,354.88 km^2^, representing a loss rate of 35.07%.

**Figure 7:**
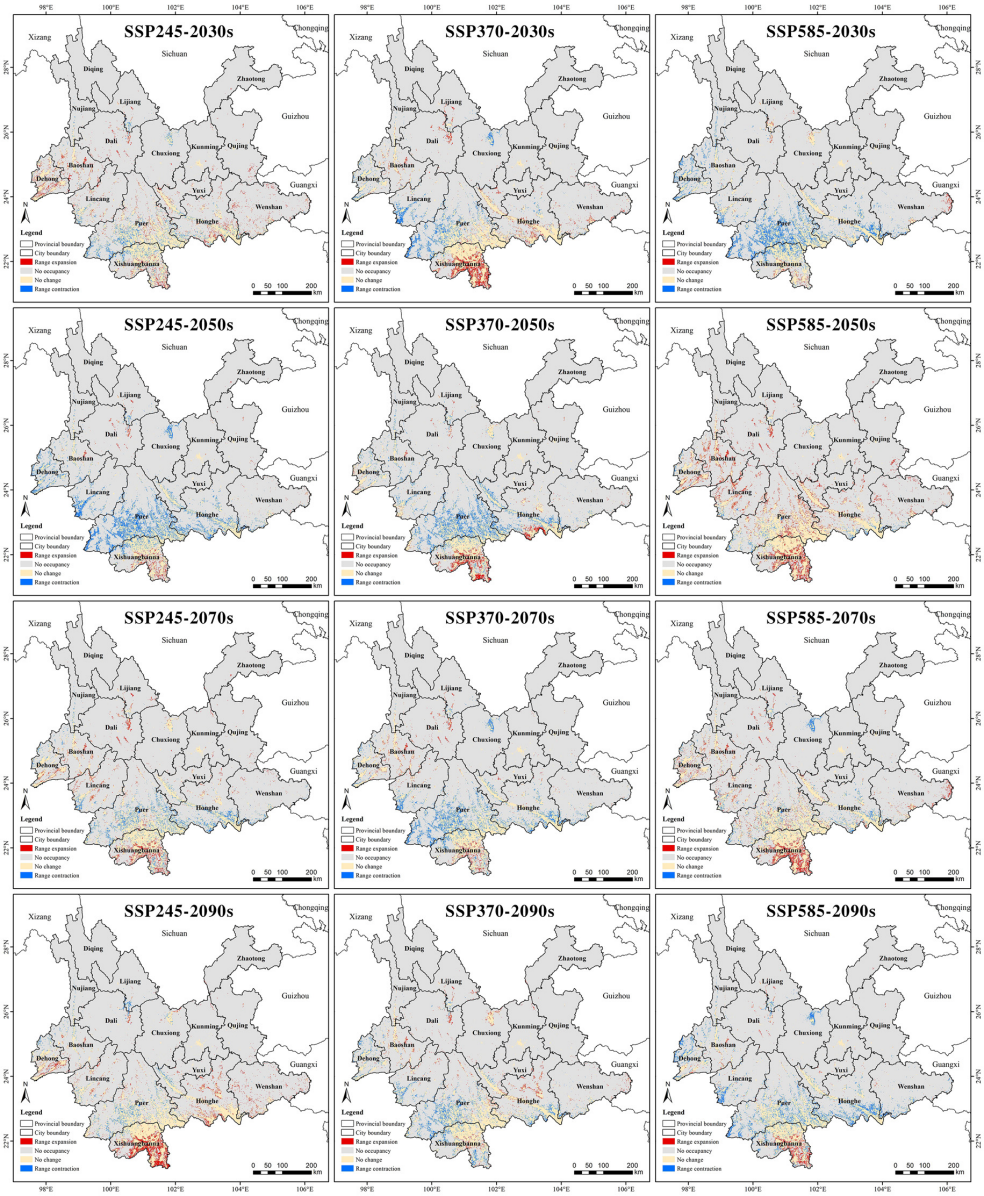
Potential habitat changes of Arabica coffee from current to future climatic conditions.

In terms of the potential suitable habitat for Arabica coffee in Yunnan Province, **Figure 7** reveals that the expanded suitable areas in each period are mainly concentrated in Xishuangbanna, while the lost land is mainly concentrated in Pu’er. Under the climate scenarios of 2040-2060-SSP2-4.5, 2040-2080-SSP3-7.0, and 2021-2040-SSP5-8.5, Pu’er has experienced a significant contraction of its suitable areas as the city where Arabica coffee is currently most suitable for growth. Conversely, Xishuangbanna which is located at a lower latitude has maintained significant growth in suitable areas under different future climate scenarios, especially evident during the periods of 2021-2040-SSP3-7.0, 2080-2100-SSP2.45, and 2040-2080-SSP5-8.5.

**Figure 8:**
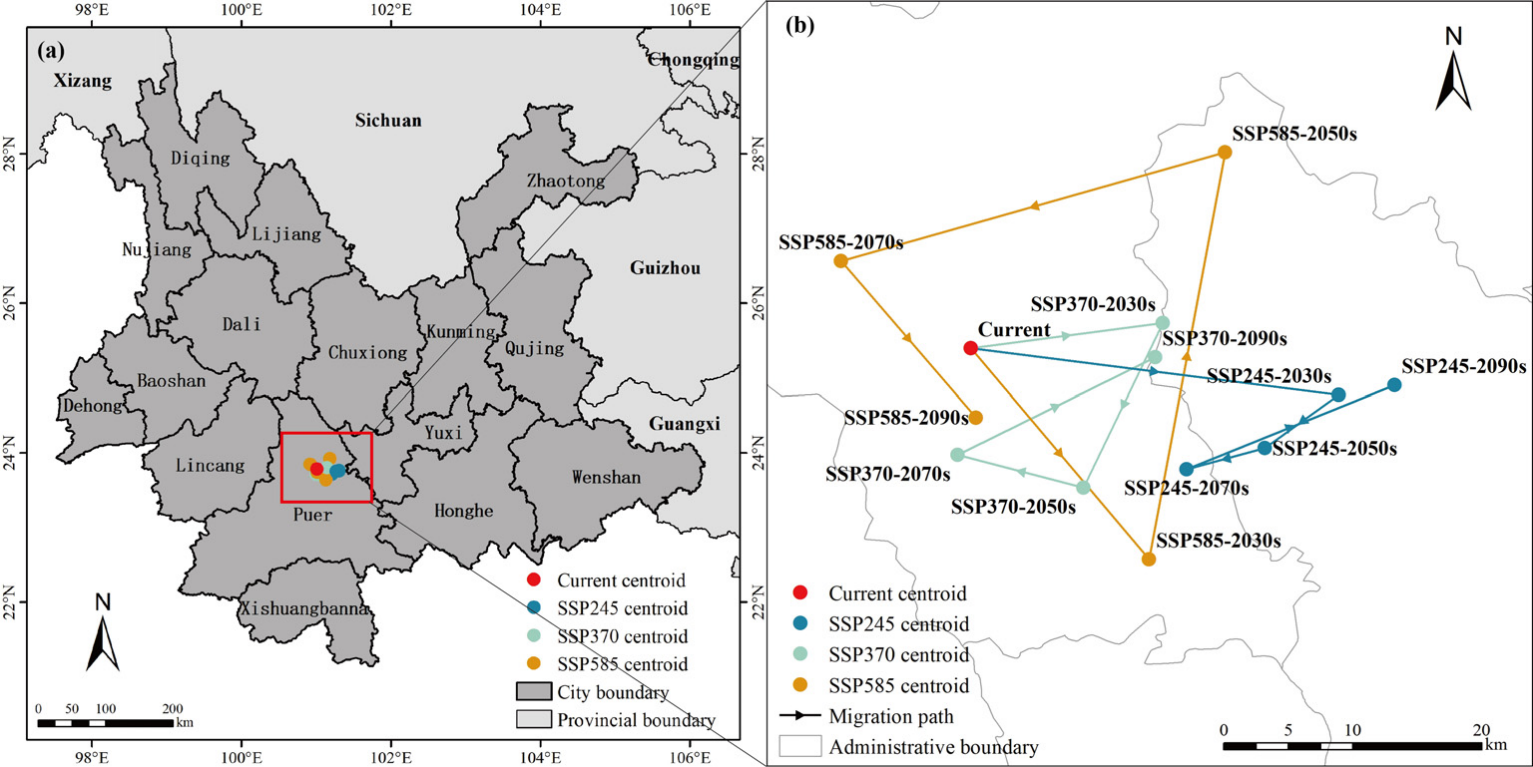
**(A)** Core distribution Changes under 12 future climate scenarios/periods. **(B)** Lines indicate the magnitude of projections over time and arrows indicate their direction.

To further investigate the response of Arabica coffee in Yunnan Province to climate change, the change in the centroid range of its potential suitable habitat under different future climate scenarios was analyzed as shown in **Figure 8**. The centroids of suitable areas are mainly concentrated in Pu’er and tend to move towards lower latitudes in most scenarios. Under the SSP2-4.5 scenario, the centroids of suitable areas generally move eastward with latitudes lower than the current centroid by distances of 26.42 km, 6.66 km, 5.82 km, and 16.17 km in different periods. In the SSP3-7.0 scenario, the trend of centroid movement is similar to that in the SSP2-4.5 scenario but the movement distance is relatively smaller. The centroid moves eastward by 13.8 km in the 2030s, then shifts southward by 13.91 km in the 2050s, westward by 9.31 km in the 2070s, and finally moves eastward by 15.95 km in the 2090s, returning to a latitude similar to the current centroid. In the SSP5-8.5 scenario, the magnitude of centroid displacement is larger compared to the other two scenarios and the trend is different. In the 2090s, the centroid moves to the south of the current centroid, with distances of 20.65 km, 31.93 km, 28.56 km, and 15.45 km in different periods. The geographic locations of centroids for each climate scenario and period are provided in **Supplementary Table S5**.

## 4 Discussion

### 4.1 Accuracy of model prediction

The default parameters of MaxEnt were initially set based on testing data from 266 species across six different geographic regions by early model developers (Merow et al., 2013). However, utilizing only default parameters may lead to overfitting and decreased accuracy in model predictions (Tracy et al., 2018). Furthermore, the complexity of the model has a significant impact on the species’ predictive ability. Studies have shown that constraining the complexity of the MaxEnt model can be achieved by using AICc parameters and adjusting the multiplier options (Warren et al., 2014).In this study, the R programming language’s kuenm package was employed to filter through 1,160 model results with various settings, including 40 multiplier levels and 29 feature combinations. Among the combinations that met the corresponding conditions, those with the smallest delta_AICc values were selected to optimize the settings. After re-modeling, improved predictions of the potential suitable habitat for Arabica coffee in Yunnan, China, were obtained compared to the predictions based on default parameters.

Although the MaxEnt model has proven its efficiency in species distribution research, studies have indicated that the accuracy of occurrence data should be considered in model usage, as it significantly impacts the model’s fit (Anderson et al., 2020). Despite the diverse sources of species distribution data used in this study and efforts to maintain their independence, inevitable influences from the quality of database records could lead to distortions and expansions in inferred environmental associations and suitable areas (Gábor et al., 2020). Additionally, there must be a temporal correspondence between the recorded occurrence data of species and biophysical variables and the biophysical variables influencing species distribution must have statistical significance. However, due to the opportunistic nature of data sampling, certain habitat conditions of species may be overly represented (Monsarrat et al., 2019). Consequently, eliminating sampling biases of variables used in the study while retaining essential ecological signals remains challenging (Franklin, 2023). Moreover, when extending the model to areas beyond the study region, selecting appropriate variables will determine the model’s fit on a larger scale, which is crucial for subsequent research considerations (Feng et al., 2019).

### 4.2 Environmental variables affecting habitat suitability of Arabica coffee in Yunan

In this study, Pearson correlation analysis and contribution ratio were used to determine the main environmental factors affecting the distribution of Arabica coffee, and ten bioclimatic variables, three topographic factors, and four soil factors were selected as independent variables for the prediction model (Avelino et al., 2005). Response curves for important environmental predictors are shown in **Supplementary Figure S8**.

Temperature is an important climatic factor affecting the growth and development of Arabica coffee. In particular, low temperatures are the most significant. In the coldest months, temperatures below 5°C can lead to leaf damage and growth cessation, while in Yunnan the average temperature of the coldest month is above this. The hottest month of the year, when coffee is in full bloom, has maximum temperatures of up to 25°C, which can increase photosynthetic rates and promote plant growth; however, high temperatures may cause coffee leaves to burn or dry out, resulting in a decrease in net photosynthesis (Zhang et al., 2021a). The wettest seasonal temperatures for potentially suitable habitat for coffee in Yunnan Province do not exceed 25°C.

Coffee growth has specific requirements for precipitation and its timing. Adequate rainfall is crucial for maintaining coffee plants, especially during critical growth stages such as flowering and fruit development (Thioune et al., 2020). Insufficient precipitation may lead to water stress, resulting in decreased quality and yield of coffee beans (Zhang et al., 2021b). Conversely, excessive rainfall during the flowering period can cause flower drop, adversely affecting the fruit set (Tavares et al., 2018). Therefore, a moderate and well-timed balance of rainfall throughout the growing season is ideal for coffee cultivation.

Altitude is an important topographical factor affecting the quality of Arabica coffee. The large temperature difference between day and night at high altitudes makes the coffee growth cycle long, which is conducive to the accumulation of nutrients, and the concentration of chlorogenic acid and fat increases with altitude (Bertrand et al., 2006), enhancing the flavor of the coffee beans. However, these areas have relatively low temperatures and precipitation, which are not favorable for coffee growth (Moguel and Toledo, 1999). Slope also affects soil depth, soil respiration, and nutrient utilization, and gentle slopes are suitable for growing Arabica coffee (Martins et al., 2019). Our findings are consistent with previous studies.

In terms of soil conditions, this study considers soil pH and aluminum saturation as the primary environmental variables affecting Arabica coffee in Yunnan. Soil pH is a crucial factor influencing the effectiveness of nutrients, with its variation directly impacting the absorption of nutrients by coffee trees. Small-seeded coffee is best suited to grow in soils with pH values ranging from 5.5 to 6.5. Aluminum (Al) toxicity is a major factor limiting crop productivity in acidic soils where aluminum ions (Al^3+^) primarily affect plant root systems, slowing their growth and development, reducing lateral root numbers, and consequently decreasing plant yield (Bazzo et al., 2013). While Arabica coffee is relatively more tolerant to aluminum compared to other varieties, most coffee-producing areas are located in acidic soils where the aluminum ion content is sufficient to impair plant development, damage root systems and restrict their ability to absorb water and nutrients, thereby affecting their growth and productivity (de A. Bojórquez-Quintal et al., 2014).

### 4.3 Impact of climate change on habitat suitability of Arabica coffee in Yunnan and adaptive strategies

The research findings indicate that the suitable habitats for Arabica coffee in Yunnan Province are primarily located in the western and southern regions, which are predominantly characterized by a subtropical climate. These regions align with the biological requirements of Arabica coffee and correspond with the actual distribution of coffee cultivation in Yunnan. Compared to current climate conditions, the area of suitable habitats is projected to decrease under most future emission scenarios. Notably, the magnitude of change is higher under high-emission scenarios, demonstrating the significant impact of global climate change on Arabica coffee cultivation in this region. Conversely, the area of sub-suitable habitats is projected to increase in most scenarios, showing a trend consistent with the overall reduction of suitable habitats, indicating that suitable habitats may degrade into sub-suitable ones.

The analysis of the geometric center (centroid) movement of suitable habitats in response to climate change reveals that under high-emission scenarios (SSP585), the centroid shift is more pronounced compared to other scenarios. The relationship between the centroid movement and the changes in suitable habitat areas over four future periods shows a trend where, during periods of habitat reduction, the centroid moves southeast, while during periods of habitat expansion, the centroid shifts towards higher latitudes or elevations in the northwest. This aligns with previous research indicating that climate warming leads to a reduction in species’ suitable habitats, often accompanied by a shift towards higher elevations (Walther et al., 2005; Adhikari et al., 2020).

Climate warming may extend the boundaries of suitable habitats for coffee into more subtropical regions (Barreto Peixoto et al., 2023; de Sousa et al., 2019), while changes in precipitation patterns, particularly in monsoon climate regions, may lead to increased rainfall (Collins et al., 2024) which could potentially expand the cultivation range for coffee in the region. Based on the contribution rates of environmental factors, the layout of the Yunnan Arabica coffee industry should consider not only the adaptation and improvement of existing coffee plantations to withstand meteorological hazards, such as droughts or prolonged low temperatures, through better fertilizer management to avoid soil acidification but also the long-term impacts of climate change. When establishing new coffee plantations in the future, careful planning should be undertaken to mitigate adverse factors. The overall layout should gradually shift towards higher elevations and latitudes.

### 4.4 Priority planting areas and strategies for Arabica coffee in Yunnan

**Figure 9:**
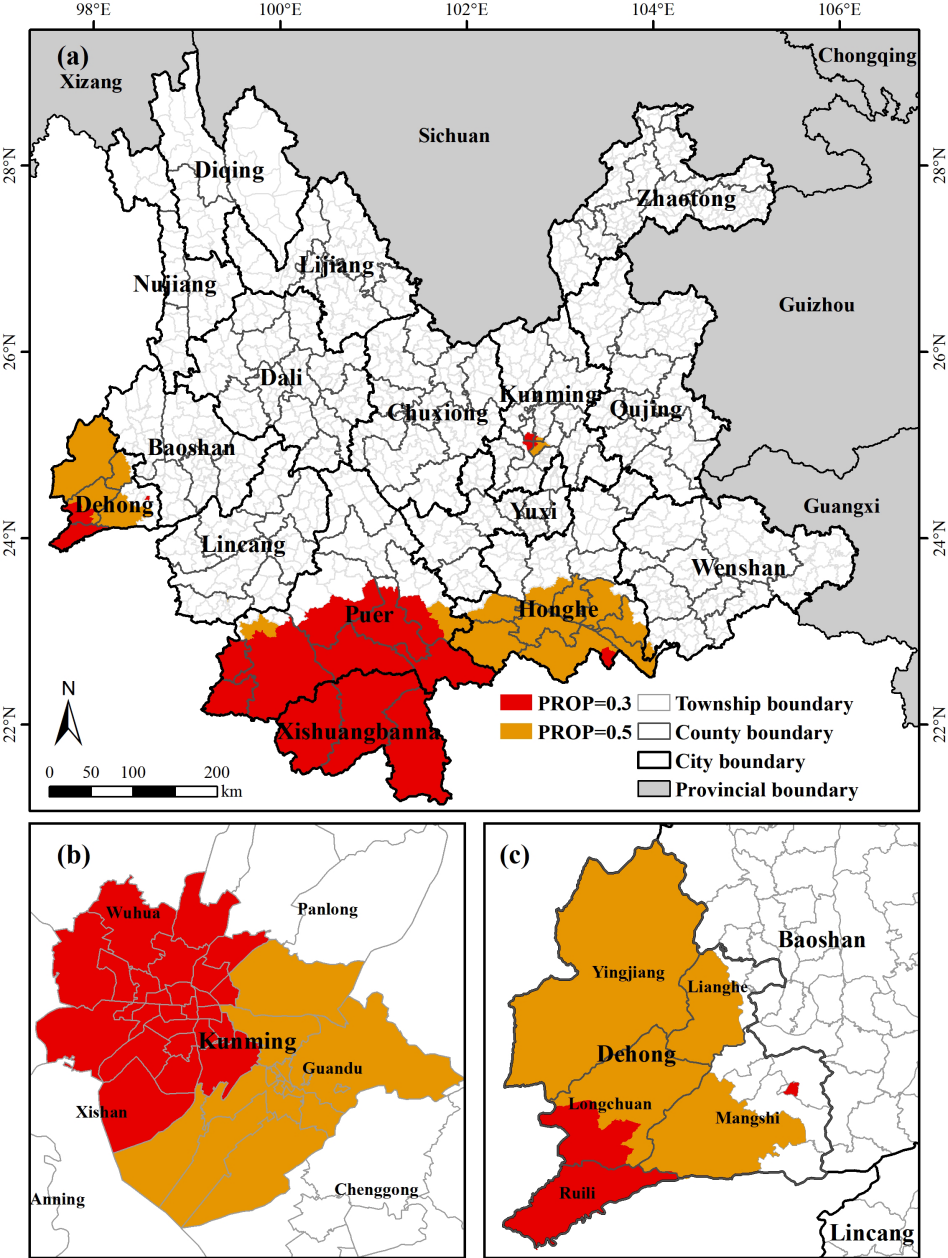
**(A)** Priority planting areas under different protection proportions; **(B)** Priority planting areas in Kunming; **(C)** Priority planting areas in Dehong.

Utilizing the Marxan model, prioritized planting areas (PPAs) for Arabica coffee were computed under different protected proportions (PROP) of 0.3 and 0.5. The simulation results were imported from ArcGIS software to generate systematic conservation plans for Arabica coffee in Yunnan province under various agricultural development goals. As illustrated in **Figure 9**, when PROP was set at 0.3, the prioritized planting areas for Arabica coffee were predominantly concentrated in Pu’er and Xishuangbanna, with some scattered distribution in Dehong, Honghe, and Kunming. A total of 133 townships were selected, covering 9.83% of all planning units. Increasing PROP to 0.5 resulted in the addition of 125 townships, which were similarly located in these three cities or prefectures, totaling 18.53% of all planning units, with the majority situated in Dehong and Honghe. These findings demonstrate the concentrated distribution of prioritized planting areas for Arabica coffee, facilitating the formulation of targeted agricultural management policies. Moreover, the congruence between these results and the predictions of the Maxent model regarding the potential suitable habitat zones for Arabica coffee in Yunnan province indicates the accuracy of the forecasts.

Systematic conservation planning is a specialized approach designed for effective decision-making in species protection. The appropriate planning units identified through systematic conservation planning represent the hotspots of Arabica coffee distribution in Yunnan. Currently, Arabica coffee studied as an economic crop is widely cultivated. The results obtained through integration with species distribution models can be used to identify priority planting areas for coffee in the region and formulate corresponding strategies. Overall, the selected PPAs mainly cover the primary production areas of Arabica coffee in Yunnan, including Pu’er, Dehong, and Xishuangbanna, where coffee has been cultivated for an extended period. According to the simulations of the MaxEnt model, these areas are suitable for coffee cultivation under current climatic conditions and are projected to remain stable in the face of future climate change scenarios. Local governments can delineate coffee planting areas based on their own climatic, topographic, and soil conditions, potentially extending the lifespan of coffee cultivation and bringing lasting economic benefits to local communities.

The study found that several planning units within the WuHua, XiShan, GuanDu, and PanLong Districts of Kunming, the capital of Yunnan Province, were included in the systematic conservation plan under two different PROP conditions. These areas are located in the economic core of Yunnan and under future climate change scenarios, the expansion or contraction of suitability in these areas is more pronounced. Therefore, large-scale coffee cultivation in these areas presents certain challenges. Research suggests that agricultural landscape trajectories are formed by the action or reaction of local communities to socio-economic and environmental driving factors as well as local processes interacting at different spatial scales (Ribeiro Palacios et al., 2013). With the burgeoning development of niche economies, many coffee-growing regions offer a variety of services such as leisure/tourism and sustainable food production, all of which have the potential to benefit producers, consumers, and the environment (Jha et al., 2014; Zhou et al., 2023). Currently, some of the world’s major coffee-producing plantations are developing coffee tourism experiences (Degarege and Lovelock, 2021). As an attractive tourist destination, Kunming is suitable for integrating coffee cultivation with cultural and tourism industries, encouraging local farmers to establish cooperatives and promote ecotourism near urban areas. Coffee tourism, as a component of rural tourism, would serve as a source for income diversification and maintenance of sustainable livelihoods (Woyesa and Kumar, 2021).

Factors related to coffee production are divided into environmental factors and agricultural genetics, with the former creating suitable conditions for coffee production and the latter determining breeding quality (Jiménez et al., 2023). This study only discusses how environmental variables determine the distribution of habitat suitability for coffee production and uses its results to simulate priority planting areas for Arabica coffee in Yunnan under different development goals. The cultivation of coffee as an economic crop requires consideration of more socio-economic factors, and the quality of coffee also determines its value to a certain extent. Research has shown that over the past fifty years, temperatures in Brazilian coffee-growing cities have increased by approximately 0.25°C per decade, while annual precipitation during flowering and ripening periods has been decreasing, leading to a decline of over 20% in coffee production in southeastern Brazil (Koh et al., 2020). For Yunnan Province, being located in mountainous areas often subjects it to more severe climate fluctuations compared to flat regions, which explains the higher climate risks in the region (Diffenbaugh and Giorgi, 2012). Compounded by rural areas’ relative lack of infrastructure and other economic development opportunities (Watson and Achinelli, 2008), the hazards induced by climate change will translate into high overall climate risks for China’s core coffee-growing regions. To achieve efficient protection and allocate resources for conservation, future cultivation, and promotion of Arabica coffee in Yunnan need to consider geographical layouts and varietal selection to mitigate adverse environmental factors and the impacts of climate change. Selecting high-quality varieties suitable for specific climates, especially in policies regulated at the county level that have a decisive impact on the towns where coffee is grown, will require increased investment to provide more agricultural services, broaden farmers’ income sources, and offer practical references for Arabica coffee cultivation.

### 4.5 Limitations and prospects

The MaxEnt model exhibits data dependence (Warren and Seifert, 2011), and its performance is influenced by the relationship between the number of constraint functions and sample size, resulting in significant computational demands during the iterative process. Insufficient distribution information may lead to predictive errors, meaning that a scarcity of distribution points or inaccuracies in environmental variables can adversely affect the model’s predictive precision (Merow et al., 2013). Future research should ensure that known distribution points within the range are as numerous and accurate as possible, and when selecting environmental variables, factors that significantly impact the target species’ distribution should be prioritized over simply including all available environmental factors.

This study only predicted the effects of climate, soil, and topography on Arabica coffee, without considering the influence of interspecific interactions such as competition and predation and other ecological factors. Consequently, the predicted potential suitable areas may deviate from the actual suitable areas. Given that different climate models and scenarios can yield varying results, the selection of future climate scenarios may affect the predictive accuracy of species’ potential distributions. An over-reliance on a single scenario or model may lead to underestimating or overestimating a species’ future viability in certain regions (Kumar, 2012). The complexity of the climate system and the indirect effects of climate change on ecosystems and species habitats such as increased frequency of extreme weather events may not be fully captured in the models.

For provincial analyses, a resolution of 30 arc-seconds is normally sufficient to capture species distribution patterns at larger scales. However, given the spatial heterogeneity of localized ecological processes (e.g., microclimate effects), the use of this resolution may ignore minor differences in habitat distribution (e.g., slope) at some smaller spatial scale. Meanwhile, the fineness of the resolution may predict suitable habitat areas inaccurately when generalizing the potential distribution to areas where the species has not been investigated.

Additionally, land use changes are often driven by complex factors, including human activities, policies, and economic considerations, making future land use policies such as the establishment of protected areas or reforestation initiatives unpredictable. This unpredictability will impact habitat availability and the conditions necessary for species survival. While the MaxEnt model typically assumes a stable ecological niche for species, in reality, species may possess a certain degree of adaptability to climate and land use changes (Pradhan et al., 2024). This adaptability may not be entirely captured by the model, potentially leading to underestimations or overestimations of a species’ future distribution capacity. Similarly, plant breeding, especially genetic engineering, may significantly alter the growth characteristics and adaptive capacity of coffee (Ngoy and Shebitz, 2020). Breeding efforts may in the future produce coffee varieties that are better adapted to extreme climate change, which in turn may affect their habitat requirements. Therefore, while the present model provides a valuable reference for assessing climate change impacts, it may have limitations in long-term predictions. Future research should incorporate ecologically relevant predictive variables, considering additional influential abiotic and biotic factors to enhance model accuracy (Comia-Geneta et al., 2024). It is also important to explore the incorporation of plant breeding progress into species distribution models to improve the accuracy of long-term climate change projections for more comprehensive spatial planning strategies.

## 5 Conclusions

In this study, MaxEnt and Marxan models were used to simulate the geographical distribution of potentially suitable habitats for Arabica coffee in Yunnan Province, China under current and future (2021-2100) climate scenarios, to identify key environmental factors affecting the distribution and to predict changes in the suitable areas and centroid migration under future scenarios. The following main conclusions were drawn.

1. The results of the potential habitat suitability evaluation of Arabica coffee in Yunnan Province using the MaxEnt model were reliable and MaxEnt predicted that the suitable and sub-suitable areas were about 4.21×10^4^ km^2^ and 13.87×10^4^ km^2^, respectively, accounting for 47.15% of the total area of the province. Among them, the suitable areas are mainly concentrated in the west and south of Yunnan, especially in Pu’er and Xishuangbanna, accounting for about 11% of the total area of Yunnan.

2. 79.1% of the cumulative contribution of Minimum temperature of the coldest month, altitude, mean temperature of wettest quarter, slope, and aluminum saturation are the key environmental variables affecting the distribution of Arabica coffee in Yunnan Province. The minimum temperature of the coldest month and the mean temperature of the wettest quarter for the survival of Arabica coffee range from about 5 to 10°C and 23 to 25°C, and the value of temperature seasonality is less than 4.5, and the annual precipitation should be more than 1200 mm. An altitude of 1200 m and a slope of less than 9° is the most suitable terrain. Soils with high aluminum content may adversely affect the growth of Arabica coffee.

3. Under the SSP3-7.0 scenario from 2021 to 2100, the area of suitable habitats decreases while the area of sub-suitable habitats increases year by year. In general, except for the small increase in the total area of potentially suitable/sub-suitable habitats between 2021 and 2040, the total area of such habitats shows a decreasing trend in all other periods, which indicates that the potential habitats of arabica coffee in Yunnan Province will show a shrinking trend in the next 80 years under this climate scenario. This shows that the potential habitat of Arabica coffee in Yunnan Province under this climate scenario will shrink, and the suitable habitat will be gradually degraded to a sub-suitable habitat. The expansion and contraction of suitable and sub-suitable habitats for Arabica coffee in Yunnan Province under different climate scenarios coexist. Under the SSP3-7.0 climate scenario, the most significant change in habitat suitability for Arabica coffee was observed and the highest degree of contraction was observed under this scenario, while the least significant change was observed under the SSP5-8.5 climate scenario, but the highest degree of expansion was observed under this scenario

4. The center of the suitable area in the optimal zone of Arabica coffee shifted eastward in the SSP2-4.5 scenario, while the trend of the center of the suitable area shifted in a direction similar to that of the SSP2-4.5 scenario but the distance of the shift was smaller in the SSP3-7.0 scenario, and the distance of the shift of the center of mass in the SSP5-8.5 scenario was larger than that in the other two scenarios.

5. Using the method of systematic conservation planning, township administrative districts as conservation units, LULC, and nature reserve data as conservation cost values, and the distribution probability of Arabica coffee as conservation characteristic values, we analyzed that the priority planting areas in Yunnan Province under the conservation objectives of 30% and 50% are Pu’er, Xishuangbanna, Honghe, Dehong, and Kunming. We put forward corresponding management policy recommendations.

## Data Availability

The raw data supporting the conclusions of this article will be made available by the authors, without undue reservation.
