# Supplementary material for: MaxEnt and Marxan modeling to predict the potential habitat and priority planting areas of Coffea arabica in Yunnan, China under climate change scenario
Source: Front Plant Sci. 2024 Nov 28;15:1471653. doi: 10.3389/fpls.2024.1471653 (PMC11635998; doi:10.3389/fpls.2024.1471653)
Supplement: Supplementary file 1 [file DataSheet1.pdf]

Supplementary Material

MaxEnt and Marxan modeling to predict the potential habitat and priority planting areas of *Coffea arabica* in Yunnan, China under climate change scenario

Xia Li, Zihao Wang*, Shaoqiang Wang, Zhaohui Qian

*** Correspondence:** Zihao Wang: zihaowang@cug.edu.cn

1 Supplementary Data

1.1 MaxEnt model

The MaxEnt model assumes that the classification model is a conditional probability distribution $P(Y|X)$, where X represents features and Y denotes the output (Phillips et al., 2006). Given a training set $(x^{(1)}, y^{(1)}), (x^{(2)}, y^{(2)}), \dots, (x^{(m)}, y^{(m)})$, where x is an n-dimensional feature vector and y is the categorical output, the relationship between input x and output y is described by a feature function $f(x, y)$, defined as

$$f(x,y) = \begin{cases} 1, & if\ x\ and\ y\ satisfy\ a\ certain\ relationship \\ 0, & otherwise \end{cases}$$

The expectation of the feature function $f(x, y)$ concerning the empirical distribution $\bar{P}(X, Y)$ is denoted as $E_{\bar{P}}(f)$, and is given by

$$E_{\bar{P}}(f) = \sum_{x,y} \bar{P}(x,y) f(x,y)$$

The expectation of the feature function $f(x, y)$ concerning the conditional distribution $P(Y|X)$ and the empirical distribution $\bar{P}(X)$ is denoted as $E_P(f)$, and is expressed as

$$E_P(f) = \sum_{x,y} \bar{P}(x) P(y|x) f(x,y)$$

If the model can be learned from the training data, we can assume these two expectations are equal, i.e.

$$E_{\bar{P}}(f) = E_P(f)$$

This equality represents the constraint for learning in the maximum entropy model. With M feature functions $f_i(x, y), (i = 1,2, \dots, M)$, there are M such constraints, implying that if there are m

samples in the training set, there are $m \times M$ constraints corresponding to the samples (Phillips and Dudík, 2008). Therefore, the definition of the maximum entropy model is as follows.

Assuming the set of models that satisfy all constraints is

$$E_{\bar{P}}(f_i) = E_P(f_i)\,, i = 1,2,\ldots,M$$

The conditional entropy on the conditional probability distribution $P(Y|X)$ is defined as

$$H(P) = -\sum_{x,y} \bar{P}(x)P(y|x)\,log\,P(y|x)$$

1.2 Marxan model

An upper limit for conservation costs is established before the execution of the objective function. When the conservation cost of the selected planning unit exceeds this threshold, a fixed penalty value is incorporated into the objective function. Throughout the process, the corresponding penalty value is monitored by adjusting various SPF values, indicating the penalty incurred when the planning scheme fails to fulfill all protection objectives. A penalty value of zero signifies that the planning scheme has successfully met all the protection goals. It is important to note that SPF is not a static parameter; different planning scenarios may necessitate the use of varying SPF values. The Boundary Length Modifier (BLM) is a parameter employed to influence the length of the protected area boundary within the planning solution. This parameter modifies the shape and boundary length of the planning solution to optimize outcomes in biodiversity conservation and natural resource management. The role of the BLM is to control the boundary length of the planning solution, thereby affecting its compactness and shape. By adjusting the BLM value, users can effectively manage the configuration of the protected area while minimizing planning costs. A larger BLM value typically results in increased planning costs; however, it also shortens the boundary length, leading to a more compact protected area (Watts et al., 2017). This compactness is beneficial for maintaining ecosystem integrity within the protected area and facilitates easier management and oversight by governmental agencies.

In this study, a sensitivity analysis method is used, which aims to find the most appropriate SPF value to optimize the effectiveness of the planning scheme by plotting a sensitivity curve. This approach helps to identify strategies that can adjust the SPF value when achieving the best results in practical applications to better meet ecological conservation needs. In this paper, concerning the established studies, first, we need to ignore the effect of the BLM value, set the BLM value to 0, and then set the SPF value in the interval of 0~10 for testing, and statistically analyze the results of the generated optimal plan, and then further search for the appropriate interval, and then carry out the sensitivity analysis. The results of the sensitivity analysis of SPF in the interval of 0 to 10 are shown in **Supplementary Figure 1** and **Supplementary Table 2**. By analyzing the results, it is found that when the SPF increases, the planning cost also increases significantly and the penalty value decreases gradually. When the SPF value is 1, the penalty value is 44.17, but the planning cost is low, only 15.47. When the SPF is 2, the penalty value decreases significantly to 0.044, and the planning cost increases to 72.45. When the SPF value continues to increase, the decrease in the penalty value is no longer significant, but the planning cost is still increasing. Therefore, we define the interval of SPF for the second sensitivity analysis as between 1 and 2.

Based on the results of the first sensitivity analysis, we narrowed the interval of SPF to between 0 and 1. We calculated the costs and penalty values of the optimal planning scheme when the SPF values were 1.0, 1.1, 1.2, 1.3, 1.4, 1.5, 1.6, 1.7, 1.8, 1.9, and 2.0, respectively, and the results of the sensitivity analysis of SPF in the interval of 1 to 2 are shown in **Supplementary Figure 2 and Supplementary Table 3**. According to the results, the SPF value is still positively correlated with the planning cost and negatively correlated with the penalty value. When the SPF value is 1.6, the penalty value is 0.167 and the planning cost is 71.29, which is an obvious inflection point in this sensitivity analysis. Considering that reducing the penalty value to 0 requires more planning costs, it is often more costly in the real situation. Therefore, we did not force the penalty value to be reduced to 0, and the SPF value in this study was set at 1.6.

We make the setting of BLM value in this paper refer to the established studies, and a sensitivity analysis is applied to the BLM value to obtain the best BLM parameters. In the sensitivity analysis of BLM values, the first task is to determine the starting value of BLM, and the starting value of BLM, BLM_0, is calculated as follows.

$$BLM_0 = \frac{COST_{max}}{BOUNDARY_{max}}$$

Where BLM_0 is the starting BLM value for the sensitivity analysis, and COSTmax and BOUNDARYmax are the maximum cost and maximum boundary length corresponding to the results of the best SPF run, respectively. Therefore, we substituted SPF = 1.6 to obtain the final $BLM_0 = 0.05$.

The formula for determining the BLM value for sensitivity analysis is as follows:

$$BLM_n = BLM_0 \times 2^n, n = 1,2,3,4, \dots N$$

Eventually, this paper determined the BLM values for sensitivity analysis as 0, 0.2, 0.4, 0.8, 1.6, 3.2, 6.4, 12.8, 25.6, 51.2, and 102.4 respectively. The BLM values were entered into the Marxan software to run respectively, and the results of the statistically optimal scenarios are shown in **Supplementary Figure 3** and **Supplementary Table 4** while BLM=1.6.

2 Supplementary Figures and Tables

2.1 Supplementary Figures

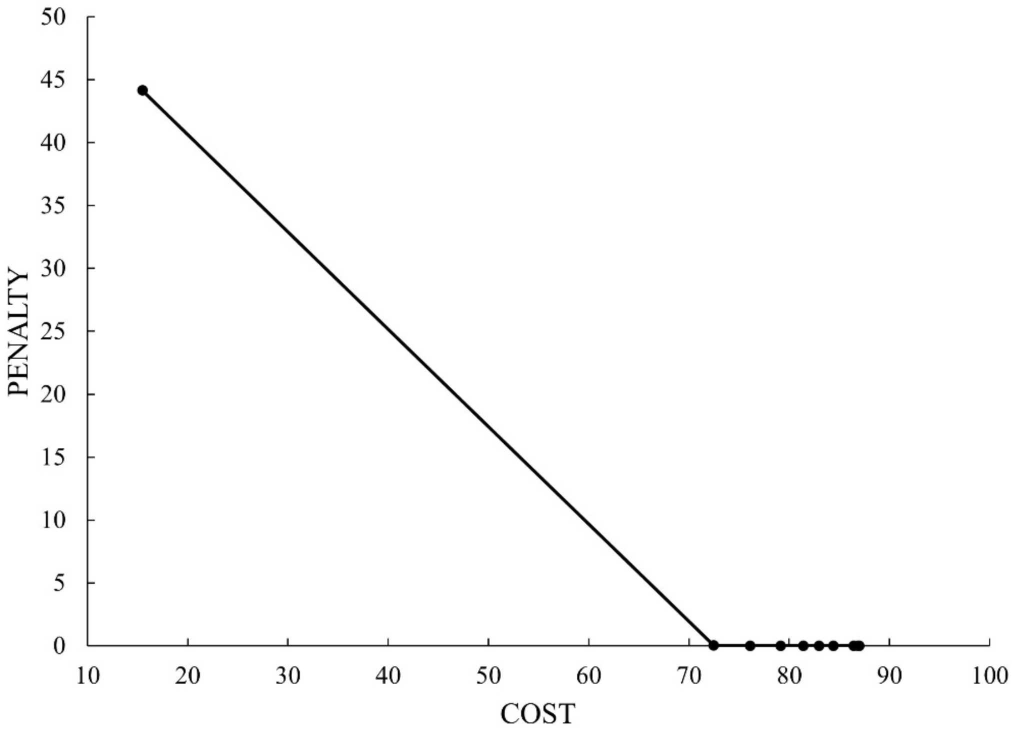

Supplementary Figure 1. Sensitivity analysis of operational results for the SPF = 0~10 interval

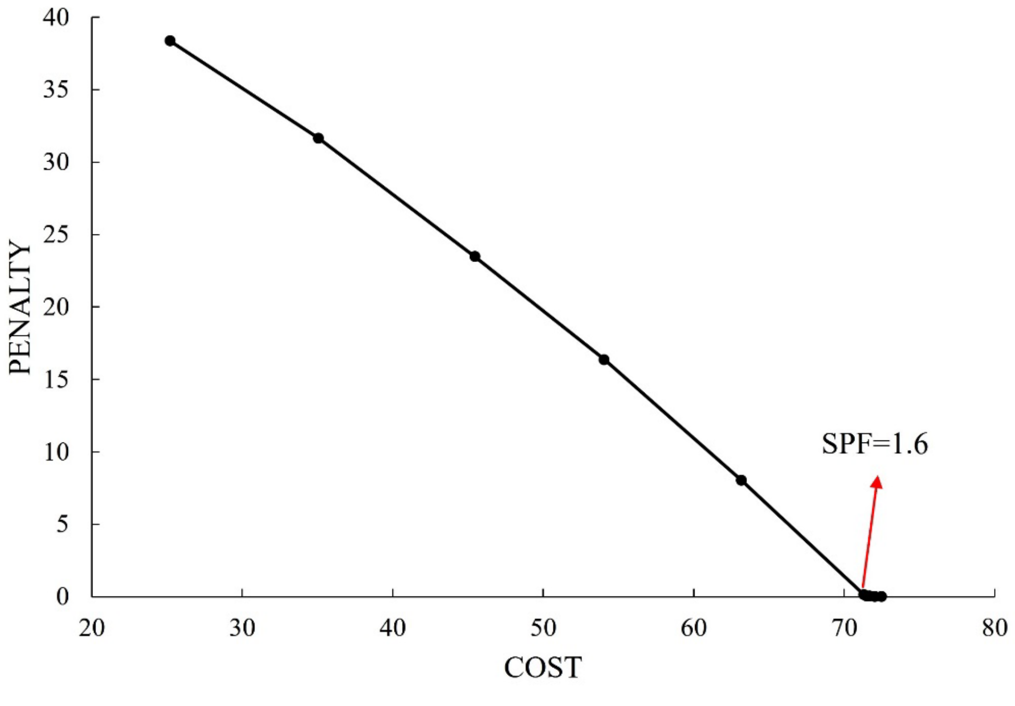

Supplementary Figure 2. Sensitivity analysis of operational results for the SPF = 1~2 interval

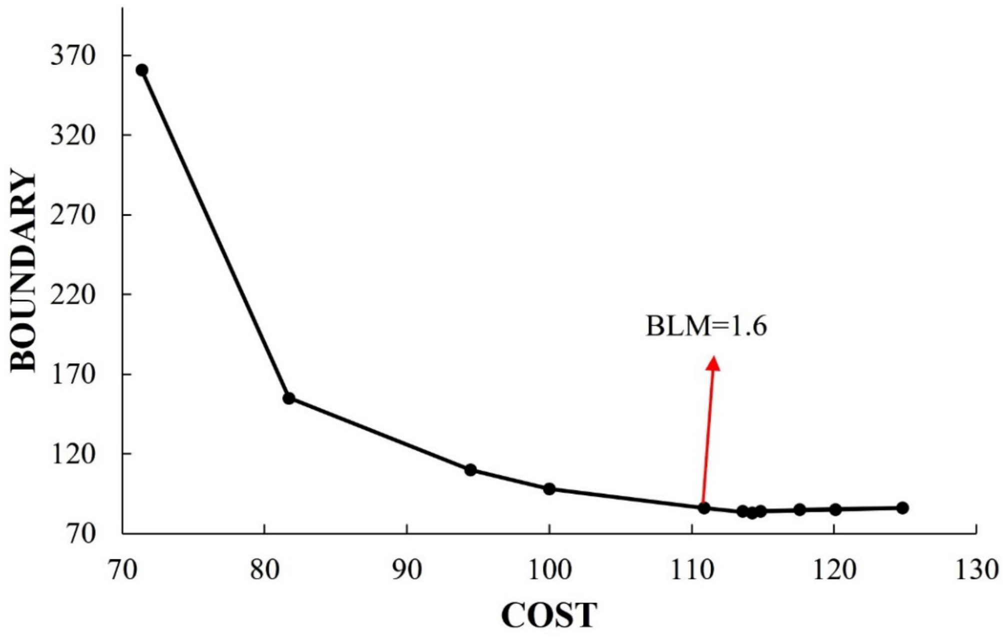

Supplementary Figure 3. Sensitivity analysis of BLM value run results

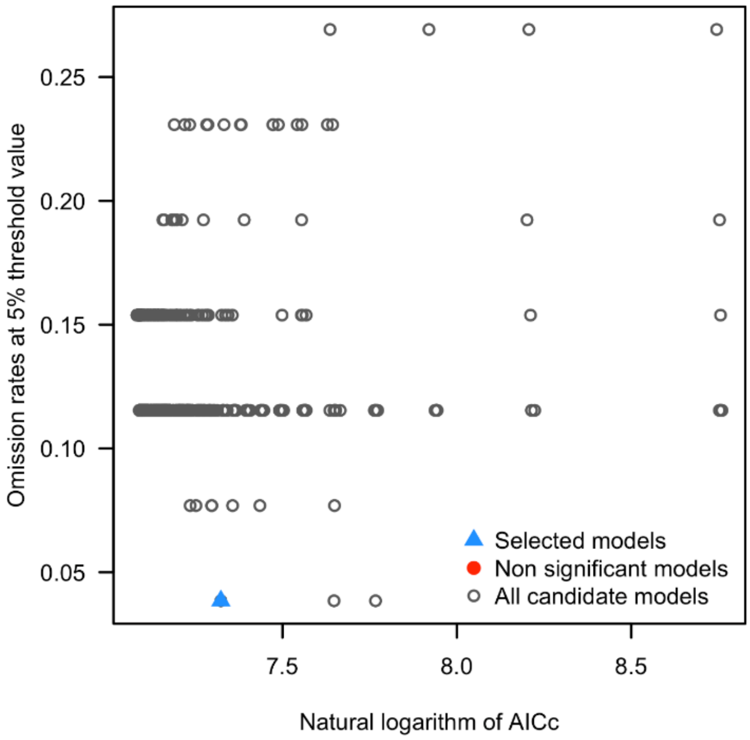

Supplementary Figure 4. Distribution of all models, non-statistically significant models, and selected models in terms of AICc and omission rate values

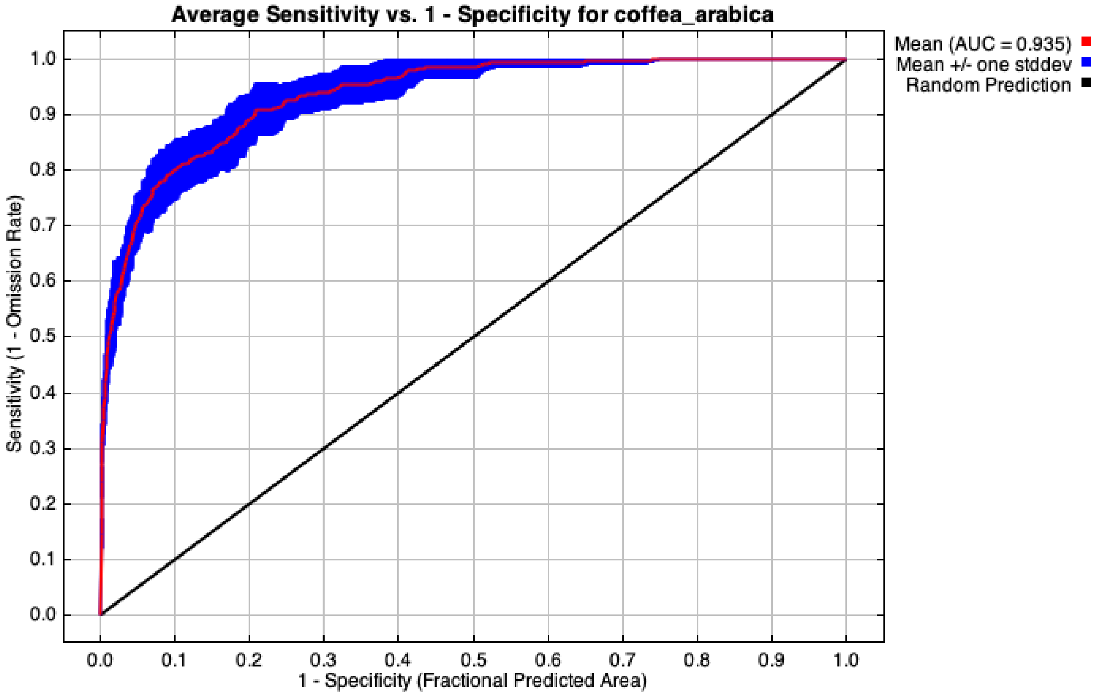

Supplementary Figure 5. Mean AUC results of ten simulations

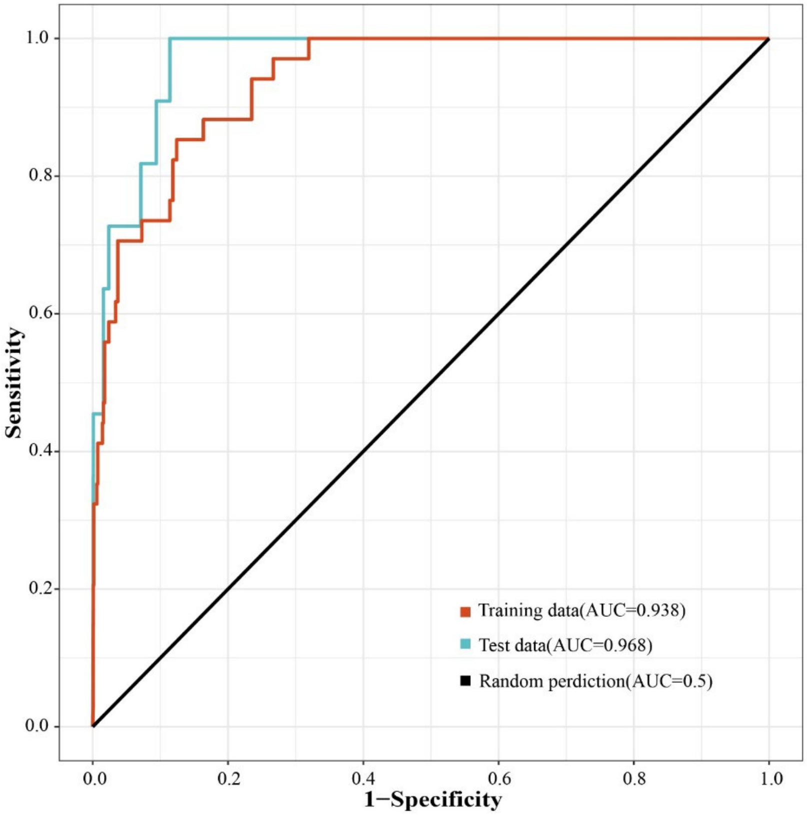

Supplementary Figure 6. Receiver operating characteristic (ROC) curve

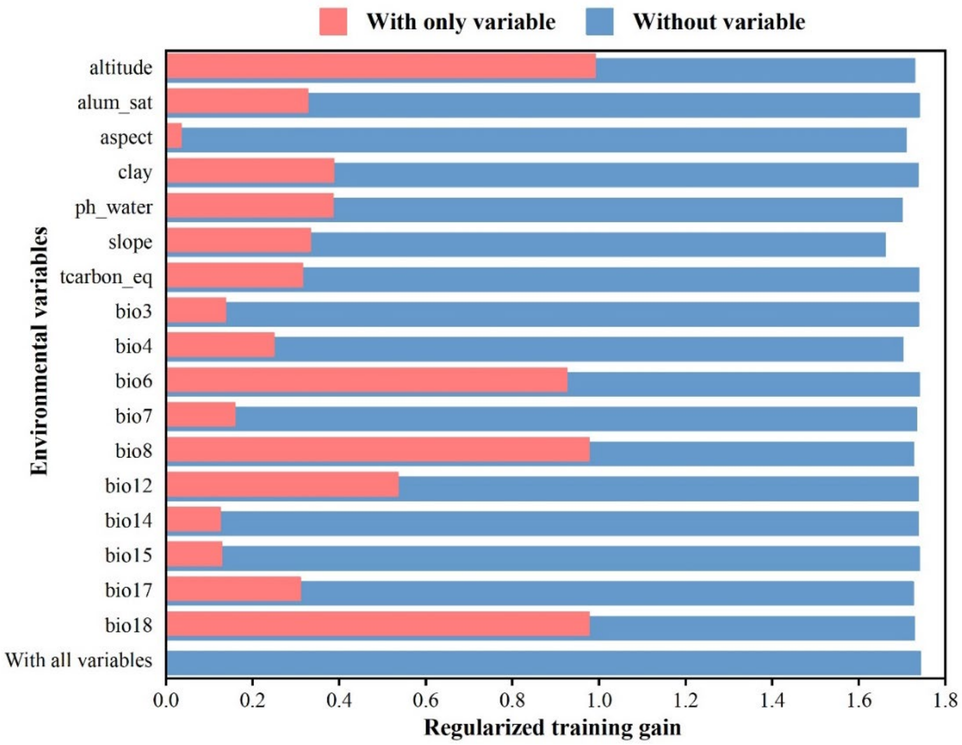

Supplementary Figure 7. Jackknife of regularized training gain for Arabica coffee

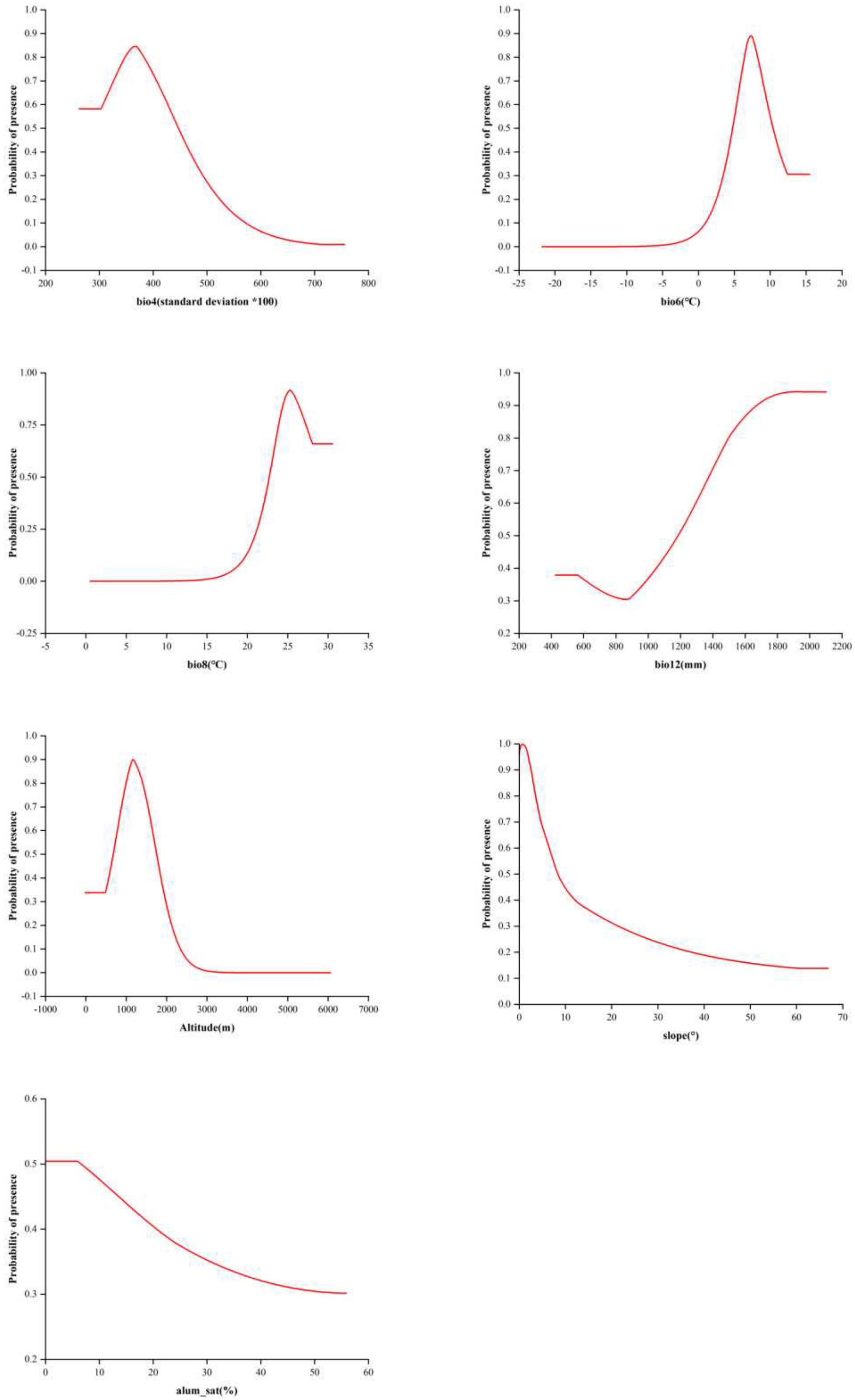

Supplementary Figure 8. Response curves for important environmental predictors

2.2 Supplementary Tables

Supplementary Table 1 Pre-experimental environmental variable results

Variable	Percent contribution	Permutation importance
bio1	30.5	0.5
slope	11.8	4.5
alum_sat	10.4	1.2
silt	6.3	0
gypsum	5.8	2.4
bio6	4.9	1
bio17	3.7	11.6
bio5	3.4	1.2
bio4	3	6.7
bio11	2.5	7.4
aspect	2.4	2.9
bio18	2.2	4.3
total_n	1.9	0.5
bio3	1.7	5.6
bio9	1.7	6.1
altitude	1.6	28.5
bio8	0.9	1.7
sand	0.8	1.1
bio14	0.8	1.4
bio15	0.7	3.3
ph_water	0.5	0.3
bio7	0.4	1.6
bio13	0.4	0.5
bio12	0.4	2.4
clay	0.3	0.3
bio2	0.3	1.5
cec_soil	0.2	0.1
bio16	0.2	0.2
bio19	0.1	0.2
tcarbon_eq	0.1	0.8
bio10	0	0
org_carbon	0	0

Supplementary Table 2 SPF=0~10 interval running result statistics

SPF	COST	PENALTY	TARGET
1	15.4742	44.1734	NO
2	72.44987	0.044057935	NO
3	76.07527	0.027464128	NO
4	79.11331	0.028229243	NO
5	81.39914	0.022501888	NO
6	82.96143	0.016888186	NO
7	84.36407	0.013353661	NO
8	86.41775	0.015931376	NO
9	86.95992	0.011736937	NO
10	86.82474	0.013239567	NO
100	93.37093	0.001513093	NO

Supplementary Table 3 SPF=1~2 interval running result statistics

SPF	COST	PENALTY	TARGET
1.1	25.1758	38.3833	NO
1.2	35.0348	31.6698	NO
1.3	45.4317	23.5013	NO
1.4	54.0321	16.3881	NO
1.5	63.1613	8.05941	NO
1.6	71.29349	0.16676678	NO
1.7	71.47214	0.082282509	NO
1.8	71.67873	0.088644498	NO
1.9	72.0262	0.04284624	NO
2	72.44987	0.044057935	NO

Supplementary Table 4 Statistics of running results for different BLM values

BLM	COST	BOUNDARY	PUs
0	71.3643	361	110
0.2	81.7117	155	106
0.4	94.455	110	112
0.8	99.9964	98	117
1.6	110.839	86	130
3.2	114.232	83	133
6.4	113.553	84	133
12.8	120.067	85	141
25.6	114.819	84	135
51.2	124.799	86	145
102.4	117.551	85	138

Supplementary Table 5 Geographic locations of centroids for each climate scenario and period

Climate scenarios	Latitude	Longitude	Migration distance (km)
Current	101° 0' 43"	23° 47' 16"	-
SSP2-4.5-2030s	101° 16' 6"	23° 45' 19"	26.42
SSP2-4.5-2050s	101° 13' 1"	23° 43' 6"	6.66
SSP2-4.5-2070s	101° 9' 44"	23° 42' 13"	5.82
SSP2-4.5-2090s	101° 18' 27"	23° 45' 44"	16.17
SSP3-7.0-2030s	101° 8' 45"	23° 48' 19"	13.80
SSP3-7.0-2050s	101° 5' 26"	23° 41' 27"	13.91
SSP3-7.0-2070s	101° 0' 10"	23° 42' 49"	9.31
SSP3-7.0-2090s	101° 8' 26"	23° 46' 54"	15.95
SSP5-8.5-2030s	101° 8' 11"	23° 38' 27"	20.65
SSP5-8.5-2050s	101° 11' 22"	23° 55' 28"	31.93
SSP5-8.5-2070s	100° 55' 18"	23° 50' 55"	28.56
SSP5-8.5-2090s	101° 0' 57"	23° 44' 23"	15.45

3 Reference

Klein C, Wilson K, Watts M, et al., 2009, Incorporating ecological and evolutionary processes into continental-scale conservation planning. Ecological Applications, 19(1): 206-217.

Johnson J A, Runge C F, Senauer B, et al., 2014, Global agriculture and carbon trade-offs. Proceedings of the National Academy of Sciences, 111(34): 12342-12347.

Phillips, S.J., Anderson, R.P., Schapire, R.E., 2006. Maximum entropy modeling of species geographic distributions. Ecological Modelling 190, 231–259. https://doi.org/10.1016/j.ecolmodel.2005.03.026

Phillips, S.J., Dudík, M., 2008. Modeling of species distributions with Maxent: new extensions and a comprehensive evaluation. Ecography 31, 161–175. https://doi.org/10.1111/j.0906-7590.2008.5203.x

Watts, M.E., Stewart, R.R., Martin, T.G., Klein, C.J., Carwardine, J., Possingham, H.P., 2017. Systematic Conservation Planning with Marxan, in Gergel, S.E., Turner, M.G. (Eds.), Learning Landscape Ecology: A Practical Guide to Concepts and Techniques. Springer, New York, NY, pp. 211–227. https://doi.org/10.1007/978-1-4939-6374-4_13
